# Sonodynamic Therapy-Based DNA Nanocarriers with Hypoxia-Inducible Factor-1α Silencing Activation for Precision Lung Cancer Therapy

**DOI:** 10.34133/bmr.0230

**Published:** 2025-08-21

**Authors:** Yuchao Cao, Shangfeng Shen, Jiahui Xiang, Yan Qiu, Jiajun Guo, Yuqing Zhang, Dairong Li, Yonghong Du

**Affiliations:** ^1^ State Key Laboratory of Ultrasound in Medicine and Engineering, College of Biomedical Engineering, Chongqing Medical University, Chongqing, 400016, China.; ^2^ Department of Respiratory and Critical Care Medicine, the First Affiliated Hospital of Chongqing Medical University, Chongqing, 400016, China.

## Abstract

As lung cancer is still the deadliest cancer worldwide, there is an urgent need for safer and more efficient therapies. This study aims to address the challenges posed by tumors in reducing the efficacy of sonodynamic therapy (SDT) through mechanisms such as hypoxia and abnormal blood vessel formations. In this study, manganese-containing DNA nanoflowers (DHA-DDF) loaded with doxorubicin (DOX) were functionalized with an AS1411 aptamer and a hypoxia-inducible factor-1α (HIF-1α) antisense sequence. The in vitro tests confirmed their stability and pH-responsive drug release properties. The combined treatment of DHA-DDF and ultrasound could induce apoptosis, inhibit the migration and invasion of Lewis lung carcinoma (LLC) cells, and down-regulate the expression of HIF-1α and VEGF in LLC cells. The in vivo studies using subcutaneous LLC in mice showed that ultrasound enhanced the tumor-targeted accumulation and penetration of DHA-DDF. The combined approach markedly reduced tumor development and extended the survival of tumor-bearing mice, effectively down-regulated the expression of hypoxia-related genes, inhibited cell proliferation, and blocked tumor angiogenesis. The programmable, biocompatible, and multifunctional nanoflowers demonstrate a notable improvement in the efficacy of SDT and provide robust tumor inhibition in both cellular and animal models. The findings highlight the potential of DNA nanotechnology in advancing innovative cancer therapies.

## Introduction

Lung cancer, the cancer with the highest mortality rate in the world [1], poses a serious threat to human life and lack effective therapeutic methods. Multidrug resistance and metastasis are the toughest problems to curing tumors [2,3]. The tumor microenvironment, including vascular abnormalities, high interstitial pressure, hypoxia, and acidity [4,5], is conducive to tumor growth while hindering drug penetration and enabling immune evasion [6–8].

Sonodynamic therapy (SDT) is a noninvasive, radiation-free, highly penetrative, and directionally precise method, which employs low-intensity ultrasound (US) to activate sonosensitizers for targeted tissue treatment [9]. It exhibits extremely promising application prospects in the field of clinical oncology. However, the limited half-life of conventional sonosensitizers in circulation, marked adverse effects, and expensive manufacturing costs pose major obstacles to their widespread use in clinical cancer [10]. Furthermore, the aberrant tumor microenvironment, which is characterized by hypoxia, acidity, and high interstitial pressure, has also been shown to pose a challenge to advanced therapies as well as traditional ones like chemotherapy and radiation therapy, thereby compromising their therapeutic efficacy [11–13].

Hypoxia is a major characteristic resulting from the abnormal metabolisms of cancer cells, which might reduce the efficacy of various therapies [11–13]. Hypoxia-inducible factor-1α (HIF-1α) is a key cytokine in eukaryotic cells that stabilizes in hypoxia conditions. This causes the expression of glucose transporter 1 (GLUT1) to be up-regulated, increasing glucose uptake and disrupting metabolite homeostasis [14–16]. These increase the efflux of chemotherapy drugs, leading to multidrug resistance [17–19]. For organizational dimension, HIF-1α stabilization in hypoxia conditions obstructs drug penetration by up-regulating the expression of vascular endothelial growth factor (VEGF) and inducing abnormal angiogenesis [20]. Thus, reducing the level of HIF-1α may improve the anticancer effects of SDT [21,22].

DNA nanoflower (DF), an organic/inorganic hybrid nanoparticle with porous, petal-like structure composed of long stranded DNA (as a shell) and pyrophosphates (as a core), has gained widespread attention owing to its versatile functions since first reported in 2012 [19,22–24]. The long stranded tortuous DNA on the surface not only is biocompatible and stable but also can be used as antisense oligonucleotide (ASO) or ribozyme to regulate the expression of genes and proteins [22,25]. Doxorubicin (DOX), a sonosensitizer [26–28] and a chemotherapeutic agent that is used commonly in tumor therapy [29,30], can bind to DNA through hydrogen bonding or electrostatic adsorption [19,31,32]. Because of these characteristics, DF can be used as nanocarriers for transporting DOX and ASO. Unlike traditional drugs, ASO acts directly at the genetic level, modulating the expression of cytokines through disruption of cellular transcription or translation processes.

**Fig. 1.**
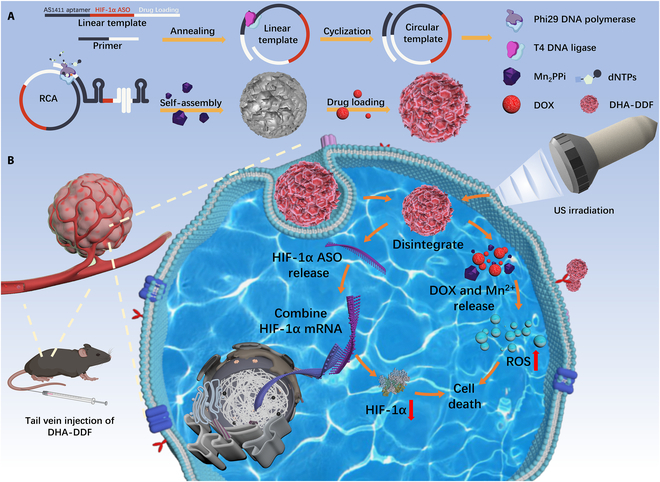
(A) Illustration of the synthesis of DHA-DDF. (B) Proposed strategy for enhanced SDT utilizing DHA-DDF.

Herein, we designed and synthesized manganese ion-based DNA nanoflowers (DHA-DFs) that were loaded with DOX, HIF-1α ASO, and AS1411 aptamer. These DFs specifically suppress HIF-1α expression of tumor cells, thereby potentiating both chemotherapy and SDT outcomes. Furthermore, by encoding the AS1411 aptamer sequence in DF, the nanoflowers are capable of active targeting, which improves drug delivery efficiency and minimizes the toxicity of DOX to normal tissues. We further suggest using the mechanical properties of US to enhance DF penetration into tumor tissue and activate DOX’s sonosensitivity, which would lead to an optimal treatment outcome, since abnormal vasculature in tumors causes inefficient material delivery. The study investigates the therapeutic impact and mode of action of DHA-DDF (DOX-loaded DF) in conjunction with US on lung cancer, potentially offering a novel approach to treatment for clinical use. The proposed technique for enhanced SDT and chemo-dynamic therapy employing the DHA-DDF nanoplatform carrier is outlined in Fig. 1, which also shows how DHA-DDF is synthesized.

## Materials and Methods

### Materials, cells, and animals

All oligonucleotide sequences and primers were synthesized and high-performance liquid chromatography-purified by Sangon Biotech Co. Ltd. (China). Stock solutions of DNA were prepared with phosphate buffer (20 mM, pH 7.0) and stored at −20 °C. T4 DNA ligase and 20-base pair DNA ladder were obtained from Takara Biomedical Technology Co. Ltd. (China). Phi29 DNA polymerase was purchased from New England Biolabs Inc. (USA). Deoxynucleotide triphosphate (dNTP) mix solution and Gel Red were purchased from Shanghai Sangon Biotech Co. Ltd. Doxorubicin hydrochloride, 1,3-diphenylisobenzofuran (DPBF), fluorescein isothiocyanate (FITC)-lectin, fluorescein-12-deoxyuridine triphosphate (FITC-dUTP), and cyanine 5-deoxycytidine triphosphate (Cy5-dCTP) were purchased from Sigma-Aldrich (USA). Dulbecco’s modified Eagle’s medium (DMEM), fetal bovine serum (FBS), and phosphate-buffered saline (PBS) were purchased from GIBCO Invitrogen Corp. Beyotime Biotechnology (China) provided deoxyribonuclease (DNase) I, agarose, 4′,6-diamidino-2-phenylindole (DAPI), Hoechst 33342, 4% PFA fix solution, 2′,7′-dichlorofluorescein diacetate (DCFH-DA), dihydroethidium (DHE), hydrogen peroxide assay kit, LysoTracker (Green), BCA protein assay kit, lipid peroxidation MDA assay kit, calcein/propidium iodide (PI) cell viability/cytotoxicity assay kit, Annexin V-FITC apoptosis detection kit, crystal violet, glucose assay kit, lactate assay kit, terminal deoxynucleotidyl transferase dUTP nick end labeling (TUNEL) assay kit, sodium dodecyl sulfate–polyacrylamide gel electrophoresis (SDS-PAGE) gel quick preparation Kit, SDS-PAGE sample loading buffer, radioimmunoprecipitation assay (RIPA) lysis buffer, protease inhibitor cocktail, bovine serum albumin (BSA), and FITC goat anti-rabbit immunoglobulin G (IgG) secondary antibody. Polyvinylidene fluoride (PVDF) membrane was purchased from Millipore System Inc. (USA). Cell counting kit-8 (CCK-8) was purchased from Dojindo (Japan). Transwell plates were purchased from Corning Incorporated (USA). Manganese dichloride and Matrigel basement membrane matrix were purchased from Shanghai Aladdin Biochemical Technology Co. Ltd. (China). The solid pimonidazole HCl (hypoxyprobe-1) and affinity-purified rabbit anti-pimonidazole antibody were purchased from Bride Biotechnology Co. Ltd. (Beijing, China). Anaero Pack was purchased from Mitsubishi Gas Chemical Company Inc. (Japan). Enzyme-linked immunosorbent assay (ELISA) kits for HIF-1α, VEGF, CD31, α-smooth muscle actin (α-SMA), tumor necrosis factor-α (TNF-α), interleukin-6 (IL-6), and interferon-γ (IFN-γ) were purchased from Jingmei Biotechnology (China). HIF-1α antibody, CD31 antibody, GLUT1 antibody, VEGF antibody, proliferating cell nuclear antigen (PCNA) antibody, α-SMA antibody, β-actin antibody, goat anti-rabbit IgG (H+L) horseradish peroxidase (HRP), and Affinity prestained protein ladder (10 to 250 kDa) were purchased from Affinity Biosciences (USA). CD31 antibody and donkey anti-goat NL557 antibody were purchased from R&D Systems (USA). FreeZol Reagent, HiScript III RT SuperMix for quantitative polymerase chain reaction (qPCR), and ChamQ Universal SYBR qPCR Master Mix were purchased from Nanjing Vazyme Biotech Co. Ltd. (China). Ultrapure water used throughout the study was obtained by using a Milli-Q ultrapure water system (18.2 MΩ⋅cm, Millipore System Inc., USA).

The murine Lewis lung carcinoma (LLC) cell line was purchased from Shanghai Fuheng Biotechnology Co. Ltd., and the human normal bronchial epithelioid cell line (16HBE) was provided by Oncology and Epigenetics Laboratory of Chongqing Medical University. All cells were cultured in DMEM supplemented with 10% FBS and 1% streptomycin–penicillin at 37 °C in a normoxic (21% O_2_) humid atmosphere or the hypoxic (1% O_2_) humid atmosphere with 5% CO_2_. The hypoxic condition was created by using an Anaero Pack System.

Male C57BL/6 mice (6 to 8 weeks old, ≈20 g) were purchased from and raised at the Animal Experiment Center of Chongqing Medical University. LLC cells (5 × 10^5^) were subcutaneously injected into the right buttock of mice to establish a subcutaneous tumor. All animals were maintained in a sterile environment and allowed free access to food and water. All procedures involving animals in this study comply with the ethical standards established by the Experimental Animal Ethics Committee of Chongqing Medical University (ethical number: 2022152).

### Preparation of the circular DNA

The DNA oligonucleotide sequences used in this study are shown in Table S1. The DHA template for synthesizing DHA-DFs consists of a sequence complementary to the drug loading sequence (blue), a sequence complementary to the AS1411 aptamer (green), and a sequence complementary to the HIF-1α antisense (yellow). The DH template for synthesizing DH-DFs does not have the sequence complementary to the AS1411 aptamer (replaced by meaningless sequences). Similarly, the DA template does not have the sequence complementary to the HIF-1α antisense, and the HA template does not have the sequence complementary to the drug loading sequence.

To obtain the circular DNA (cDNA), phosphorylated DNA template (1 μM) and corresponding primer (1.5 μM) were hybridized in 1× T4 DNA ligase reaction buffer (66 mM tris–HCl, 6.6 mM MgCl_2_, 10 mM dithiothreitol, and 0.1 mM adenosine triphosphate, pH 7.6) by heating at 95 °C for 5 min and then allowed to gradually cool to room temperature. Subsequently, T4 DNA ligase (17.5 U/μl) was added, and the mixture was placed at 16 °C overnight. Finally, the reaction was heated to 65 °C for 10 min to inactivate T4 DNA ligase.

### Preparation of DFs

The DFs were synthesized by the rolling circle amplification (RCA) reaction. The cDNA (0.5 μM), Phi29 DNA polymerase (1 U/μl), and dNTP mixture (8 mM) were incubated in Phi29 DNA polymerase reaction buffer (50 mM tris–HCl, 10 mM MnCl_2_, 4 mM dithiothreitol, and 10 mM ammonium sulfate) at 30 °C for 8 h. Then, the reaction was terminated by heating at 65 °C for 10 min to inactivate Phi29 DNA polymerase. The DFs were washed 3 times with ultrapure water by centrifugation at 10,000 rpm for 10 min and then stored at 4 °C.

DOX was mixed with DFs and incubated at room temperature for various durations to allow for the formation of DDFs. DHA-DFs, DH-DFs, DA-DFs, and HA-DFs were prepared by using the corresponding templates shown in Table S1. FITC-dUTP or Cy5-dCTP was added to the RCA reaction system to create FITC-labeled DFs (FITC-DFs) or Cy5-labeled DFs (Cy5-DFs).

### Characterization of DFs

The molecular weight of the template, primer, circularized product, and RCA production were confirmed by 2% agarose gel electrophoresis. The external morphology and internal structure of DHA-DFs were observed by scanning electron microscopy (SEM; Hitachi, Japan) and transmission electron microscopy (TEM; Hitachi, Japan). Then, the average size and zeta potential of DHA-DFs were detected by dynamic light scattering (DLS; Malvern Instruments, UK). Additionally, the elemental compositions of DHA-DFs were analyzed by energy-dispersive x-ray spectroscopy (EDS; Hitachi, Japan).

### Determination of 

DOX concentration and loading capabilityThe RCA amplification efficiency was calculated by measuring the consumption of dNTPs in the RCA reaction. The copies of the DNA could be calculated by the following equation:

$$\mathrm{DNA}\;\text{copies}=\left(2\times 4-{\mathrm{OD}}_{260}/\varepsilon \times 100\right)\times 1000/\left(N_{\text{template}}\times 0.6\right)$$ (1)

where OD_260_ (optical density at 260 nm) represents the absorbance of the supernatant at λ = 260 nm, ε represents the extinction coefficient of dNTP, and *N*_template_ represents the number of nucleotides in the template. The method for calculating the replication efficiency of other DFs was similar. The calculation details can be seen in Table S2.

To calculate the loading capability of DOX in DFs, DOX (1 mg/ml) was mixed with DHA-DFs or HA-DFs, then incubated at room temperature for various durations, and centrifuged at 10,000 rpm for 10 min. The free DOX in the supernatant was quantified by measuring the absorbance at 233 nm using ultraviolet–visible (UV–vis) spectrophotometry (UV-2600 SHIMADZU, Japan). The amount of DOX loaded in DHA-DFs and HA-DFs was calculated by subtracting the amount of DOX in the supernatant from the total amount of DOX. The calculation details can be seen in Table S3.

### Investigation of the stability and pH responsibility

The stability of DHA-DFs in biological media was studied using native polyacrylamide gel electrophoresis (PAGE) and dynamic light scattering (DLS). The DHA-DDFs were separately incubated with PBS (pH 7.4), DMEM (with 10% FBS and 5 U/ml DNase I, pH 7.4), and MES buffer (pH 5.5). The samples (10 μl) were gathered at different time points for 2% agarose gel electrophoresis, DLS, or SEM measurement after 3 washes. The concentration of DOX in the supernatant was detected by UV–vis spectrophotometry. The concentration of Mn^2+^ in the supernatant was detected by inductively coupled plasma optical emission spectrometry (ICP-OES).

### ^1^O_2_ catalytic ability of DFs

^1^O_2_ was the main reactive oxygen species (ROS) formed during SDT. ^1^O_2_ production was detected by DPBF. DPBF solution (1 mg/ml) and 3% H_2_O_2_ solution were mixed with deionized water, free DOX, MnCl_2_, or DHA-DDFs (with the final concentration of 2 μg/ml DOX), and then the mixing solution was irradiated continuously with ultrasonic irradiation (1 MHz, 1 W/cm^2^). The amount of the ^1^O_2_ production was measured by DPBF absorption value at 410 nm using a UV–vis spectrophotometer. The consumption of DPBF was calculated by the following formula:

$$\text{DPBF consumption}\;\left(\%\right)=\left[\left({\mathrm{OD}}_{\text{t0}}-{\mathrm{OD}}_{\mathrm{tx}}\right)/{\mathrm{OD}}_{\text{t0}}\right]\times 100\%.$$ (2)

OD_t0_ represents the absorbance value of 410 nm at minute 0, and OD_tx_ represents the absorbance value of 410 nm at minute *x*.

### Measuring intracellular levels of H_2_O_2_ or MDA

LLC cells were inoculated in 6-well culture plates at 1 × 10^6^ cells/well density and grouped as follows: (I) PBS (control), (II) DOX, (III) DA-DDF, (IV) DHA-DDF, (V) PBS + US, (VI) DOX + US, (VII) DA-DDF + US, and (VIII) DHA-DDF + US. After 24 h of cultivation, cells were treated with PBS, DOX, DA-DDF, or DHA-DDF (equal to 2 μg/ml DOX). Following 4-h incubation, the treatment medium was aspirated, and cells were gently washed twice with PBS. After complete removal of PBS, fresh serum-free medium was added to each well. The cells of US treatment groups were irradiated with US (1 MHz, 1 W/cm^2^) for 3 min. At 24 h post-treatment, both adherent cells and culture supernatants were harvested for subsequent analysis. Cells were collected by trypsin–EDTA digestion, while supernatants were centrifuged to remove cellular debris.

Treated LLC cells (1 × 10^6^) were cracked by cracking solution and centrifuged at 12,000*g* for 10 min to obtain the supernatant as samples for measuring the levels of H_2_O_2_ or MDA.

### Analysis of intracellular ^1^O_2_ and O^2−^ generation

LLC cells were seeded in confocal laser scanning microscopy (CLSM) dishes and treated with the following conditions: (I) PBS (control), (II) free DOX, (III) DA-DDF, (IV) DHA-DDF, (V) US alone (US), (VI) DOX + US, (VII) DA-DDF + US, and (VIII) DHA-DDF + US. The cells were incubated with culture medium containing PBS, DOX, DA-DDFs, or DHA-DDFs (equal to 2 μg/ml DOX) for 4 h and then incubated with culture medium containing DCFH-DA probes (5 μM) or DHE probe (5 μM) in the dark for another 1 h. After washing with PBS, the cells in the US-related groups were exposed to the ultrasonic radiation (1 MHz, 1 W/cm^2^) for 3 min in the dark. Then LLC cells were observed by CLSM. For flow cytometry analysis, LLC cells were inoculated into 6-well plates and incubated for 24 h and then treated according to the above treatment methods, and intracellular fluorescence intensity was detected by flow cytometry.

### Colocalization analysis

LLC cells (nucleolin-positive cells) and 16HBE cells (nucleolin-negative cells) were seeded in confocal dishes (1 × 10^5^ cells per dish). After 24 h of incubation, the old medium was removed and replaced with DMEM containing FITC-DHA-DDF or FITC-DH-DDF (equivalent to 4 μM DOX) to continue incubation for 3 h. To observe the cellular uptake, LLC cells were incubated with DHA-DDF for 5 h and LysoTracker (Green) for 1 h. At the end of the incubation, the confocal dishes were washed 3 times with PBS, followed by treatment with 4% paraformaldehyde fixative for 10 min and DAPI staining for 8 min. Each group contains 3 parallel groups and observed by CLSM (Nikon A1, Japan) or flow cytometry (Beckman Coulter Inc., USA).

### Cytotoxicity and apoptosis assay

The cell viability in vitro was tested using the CCK-8 assay to analyze the cytotoxicity of different samples. LLC cells were inoculated in 96-well culture plates for 24 h under normoxia (21% O_2_) or hypoxia (1% O_2_). For chemotherapy, the cells were treated with serum-free medium containing DOX, DA-DDF, or DHA-DDF (DOX with final concentration of 0.0625, 0.125, 0.25, 0.5, 1, 2, 4, 8, 16, 32, and 64 μg/ml) for 4 h, and then US-related groups received US irradiation (1 MHz, 1 W/cm^2^, 3 min) (US extracorporeal noninvasive therapeutic instrument developed by Chongqing Ronghai Engineering Research Center of Ultrasound Medicine). The cells were cultured for an additional 24 h and washed with PBS, and then 100 μl of the medium containing 10% CCK-8 reagent was added to each well in the 96-well plate, followed by a 2-h incubation. The absorbance value of all samples at 450-nm wavelength was detected using a multifunctional ELISA reader to determine the cell activity. The cell activity was calculated by the following formula:

$$\begin{array}{c} \text{Cell viability}\;\left(\%\right)=\left({\mathrm{OD}}_{\text{sample}}–{\mathrm{OD}}_{\text{blank}}\right)/\\ \left({\mathrm{OD}}_{\text{control}}–{\mathrm{OD}}_{\text{blank}}\right)\times 100\% \end{array}$$ (3)

In addition, the same samples in vitro on LLC cells under hypoxia were observed by CLSM (Nikon A1, Japan) stained with calcein-AM/PI or detected by flow cytometry stained with Annexin V/PI. Subsequent in vitro experiments were conducted under hypoxic conditions.

### Wound healing assay

LLC cells were cultured in 6-well plates until the cell density reached 90% to 100%; the confluent cell monolayer was scratched using a sterile pipette tip. The cells were treated with the following conditions: (I) PBS (control), (II) free DOX, (III) DA-DDF, (IV) DHA-DDF, (V) US alone (US), (VI) DOX + US, (VII) DA-DDF + US, and (VIII) DHA-DDF + US. The LLC cells were incubated with culture medium containing PBS, DOX, DA-DDFs, or DHA-DDFs (equal to 2 μg/ml DOX) for 4 h. The cells in each group were washed with PBS, replaced with a new serum-free culture medium, and continued to culture for 24 h. The scratching area was photographed at 0 and 24 h post-treatment to evaluate the migration ability of LLC cells.

### Transwell assay

The Matrigel was diluted with precooled DMEM medium, added to the upper chamber, and solidified at 37 °C. LLC cells were co-incubated with PBS, DOX, DA-DDF, and DHA-DDF for 4 h, and then cells in the US-related groups received US irradiation. After that, LLC cells from each treated group were resuspended in serum-free medium to adjust the cell density to 5 × 10^4^ cells/ml. Cell suspensions (300 μl) were added to the upper chamber, and 800 μl of medium containing 20% FBS was added to the lower chamber and incubated at 37 °C for 24 h. Finally, the cells were fixed with precooled methanol before staining with a crystal violet. After washing and drying, the cells were observed and counted under the microscope.

### Detection of the levels of glucose and lactic acid in culture medium

The obtained culture medium was tested for residual glucose content using a glucose detection kit based on the O-toluidine method. The lactate levels in culture medium were detected using a lactate detection kit following the manufacturer’s instructions.

### Western blotting and real-time qPCR

The proteins of the cells after treatment were harvested using RIPA lysis buffer and centrifuged at 10,000*g* for 10 min at 4 °C. The supernatants were collected, separated by SDS-PAGE, and then transferred to the PVDF (0.45 μm) membrane. After blocking in a solution of 5% BSA, the membranes were washed and then incubated with primary antibodies against HIF-1α, CD31, GLUT1, VEGF, and β-actin overnight at 4 °C. Next, the membrane was incubated with secondary antibody, immersed in an electrochemiluminescence luminescent solution, and detected using a Bioanalytical Imaging System (Azure Biosystems Inc.).

Total RNA of the cells after treatment was extracted with a FreeZol Reagent according to the manufacturer’s instructions. The cDNA was synthesized from 1 μg of RNA using the HiScript III RT SuperMix for qPCR. qPCR was performed using the ChamQ Universal SYBR qPCR Master Mix. Relative gene expressions were calculated using the ΔΔ*C*T method.

### In vivo fluorescence imaging

Cy5-DHA-DFs or Cy5-DH-DFs (150 μl per mouse) were injected into the tumor-bearing mice via the tail vein. At 0, 2, 4, 8, 12, 24, and 48 h post-injection, fluorescence images of live mice were obtained by LB 983 NC320 animal imaging system. After 48 h of administration, the mice were sacrificed, the tumor tissues and main organs (hearts, livers, spleen, lung, kidney) were harvested, and fluorescence signals were detected.

### Pharmacokinetic study

Sprague–Dawley rats (females, *n* = 3) were intravenously given free DOX or DHA-DDF. Blood samples were collected from the eye sockets at 0.25, 0.5, 1.0, 2, 4, 8, 24, and 48 h after injection. Acetonitrile was added to the supernatant plasma. After volatilization, dimethyl sulfoxide was added, and drug concentrations were determined by UV–vis spectrometry.

### In vivo drug biodistribution

LLC mice were intravenously injected with DHA-DDF to investigate in vivo distribution. After drug injection, heart, liver, spleen, lung, kidney, and tumor tissues were collected at 12, 24, and 48 h and then weighed, homogenized, and lysed. Trichloromethane/isopropyl alcohol (3:1 v/v) was added for centrifugation and extraction, and drug concentrations were measured by UV–vis spectrometry after volatilization of the organic layer.

### In vivo antitumor efficacy

When the tumor volumes reached 100 to 150 mm^3^, all C57BL/6 tumor-bearing mice were randomly divided into 8 groups: (I) PBS (control), (II) free DOX, (III) DA-DDF, (IV) DHA-DDF, (V) US, (VI) DOX + US, (VII) DA-DDF + US, and (VIII) DHA-DDF + US. Mice with different treatments were administered by injecting various medications into the mice’s tail veins. After 24 h of administration, mice in the US-treated group were irradiated with US (1 MHz, 1 W/cm^2^, 3 min), and the other groups were treated with sham irradiation. The weight and tumor volume of mice were monitored every 2 d, and tumor volume was figured out as follows:

$$\text{Tumor volume}\;\left({\mathrm{mm}}^{3}\right)=\left(\text{Length}\times {\text{Width}}^{2}\right)/2.$$ (4)

$$\begin{array}{c} \text{Relative body weight}\;\left(\%\right)=\\ \left({\text{Weight}}_{\text{today}}/{\text{Weight}}_{\text{first}\;\mathrm{day}}\right)\times 100\% \end{array}$$ (5)

$$\text{Survival rate}\;\left(\%\right)=\left({\text{Number}}_{\text{alive}}/{\text{Number}}_{\text{total}}\right)\times 100\%$$ (6)

At the end of treatment, tumors, hearts, livers, spleens, lungs, and blood of mice from each group were harvested for subsequent experiments.

### Histology examination and TUNEL assay

Paraffin-embedded sections of tumor and major organs (heart, liver, spleen, lungs, kidneys) were cut at 4-μm thickness for H&E staining. Apoptosis was evaluated by TUNEL immunofluorescence (green) with DAPI counterstain (blue).

### Immunohistochemistry and immunofluorescence staining

Tumor-bearing mice were dissected after 24-h treatments. The tumor tissues were fixed in 4% paraformaldehyde solution and embedded in paraffin. Then, the tumor tissues, sliced at 4-μm thickness, were prepared for staining with PCNA, pimonidazole, HIF-1α, VEGF, PCNA, CD31, and α-SMA antibodies. Hypoxia level was quantified by the HIF-1α-positive area in the percent of tumor area. The percentage of vessels covered by pericytes was quantified by calculating the α-SMA-positive area in the percent of CD31-positive area. Microvessel density was quantified by analysis of the CD31-positive area in the percent of tumor area.

### In vivo perfusion analysis

LLC tumor-bearing mice were randomly grouped (*n* = 3 mice per group) and received various treatment. After 24 h, mice received FITC-lectin (1 mg/ml, 100 μl) injection via tail vein. After 30 min, mice were sacrificed and the tumor tissues were collected, sectioned (thickness, 10 μm) onto slides, and double-stained with anti-CD31 antibody and DAPI. Vascular perfusion was determined by calculating FITC-lectin-positive area/CD31-positive area.

### Detection of tumor hypoxia degree

LLC mice were divided into 2 groups (*n* = 3): the control group and the DHA-DDF + US group. After anesthetization, tumor tissues from both groups were scanned by photoacoustic (PA) imaging system (Vevo LAZR, Canada), and blood oxygen saturation (sO_2_) levels were calculated using the following equation:

$${\mathrm{sO}}_{2}\;\left(\%\right)=M\left({\mathrm{HbO}}_{2}\right)/\left[M\left(\mathrm{Hb}\right)+M\left({\mathrm{HbO}}_{2}\right)\right]\times 100\%.$$ (7)

### In vivo toxicity analysis

The blood samples were taken from healthy C57BL/6 mice, and the samples were washed with saline and centrifuged to collect the red blood cells (RBCs). Then, 100 μl of RBC suspension was evenly mixed with DOX or DHA-DDFs (0.01, 0.1, 1, 2.5, 5, 10, 20 mg/l) for 4 h at 37 °C. RBCs incubated with saline served as a negative control, while those with deionized water served as a positive control. After incubation, the samples were centrifuged at 2,000 rpm for 10 min, then tubes were photographed, and the absorbance value of the supernatant at 540 nm was measured by multifunctional enzyme-linked immunosorbent assay (ELISA) reader. The hemolysis rate (%) was calculated according to the following equation:

$$\begin{array}{c} \text{Hemolysis rate}\;\left(\%\right)=\left({\mathrm{OD}}_{\text{sample}}–{\mathrm{OD}}_{\text{negative}}\right)/\\ \left({\mathrm{OD}}_{\text{positive}}–{\mathrm{OD}}_{\text{negative}}\right)\times 100\%. \end{array}$$ (8)

Histopathological analysis of major organs (heart, liver, spleen, lungs, and kidneys) was performed using H&E staining to investigate the toxicity of various antitumor treatments on normal tissues at 15 and 30 d post-treatment. In addition, following various treatments, blood samples were collected from the eye sockets on days 7, 15, and 30 for analysis of blood biochemical markers, including alanine aminotransferase (ALT), aspartate aminotransferase (AST), blood urea nitrogen (BUN), creatinine (CREA), creatine kinase (CK), and total bilirubin (TBIL).

### ELISA

The level of HIF-1α, VEGF, CD31, α-SMA, TNF-α, IL-6, and IFN-γ in serum was detected by using mouse HIF-1α, VEGF, CD31, α-SMA, TNF-α, IL-6, and IFN-γ ELISA kit following the instructions provided in the manual.

### Ex vivo tumor penetration

When the tumor volume reached 100 to 150mm^3^, the mice were euthanized, and the tumor tissue was completely stripped and immersed in DHA-DDF suspension for 3 min. Then, the US treatment group was irradiated with US (1 MHz, 1 W/cm^2^, 3 min), while the control group was treated with sham irradiation. The frozen sections of these samples were stained with DAPI and observed by CLSM.

### Statistical analysis

At least 3 results were yielded for each experiment, all data were expressed as mean ± standard deviation (SD), and statistical analysis was performed using GraphPad Prism version 8.0 for Windows. Significant differences among groups were analyzed using a one-way analysis of variance (ANOVA), and differences for individual groups were determined using Student’s *t* test. The results were regarded as a significant difference when *P < 0.05, **P < 0.01, and ***P < 0.001.

## Results and Discussion

### Design and synthesis of DF

**Fig. 2.**
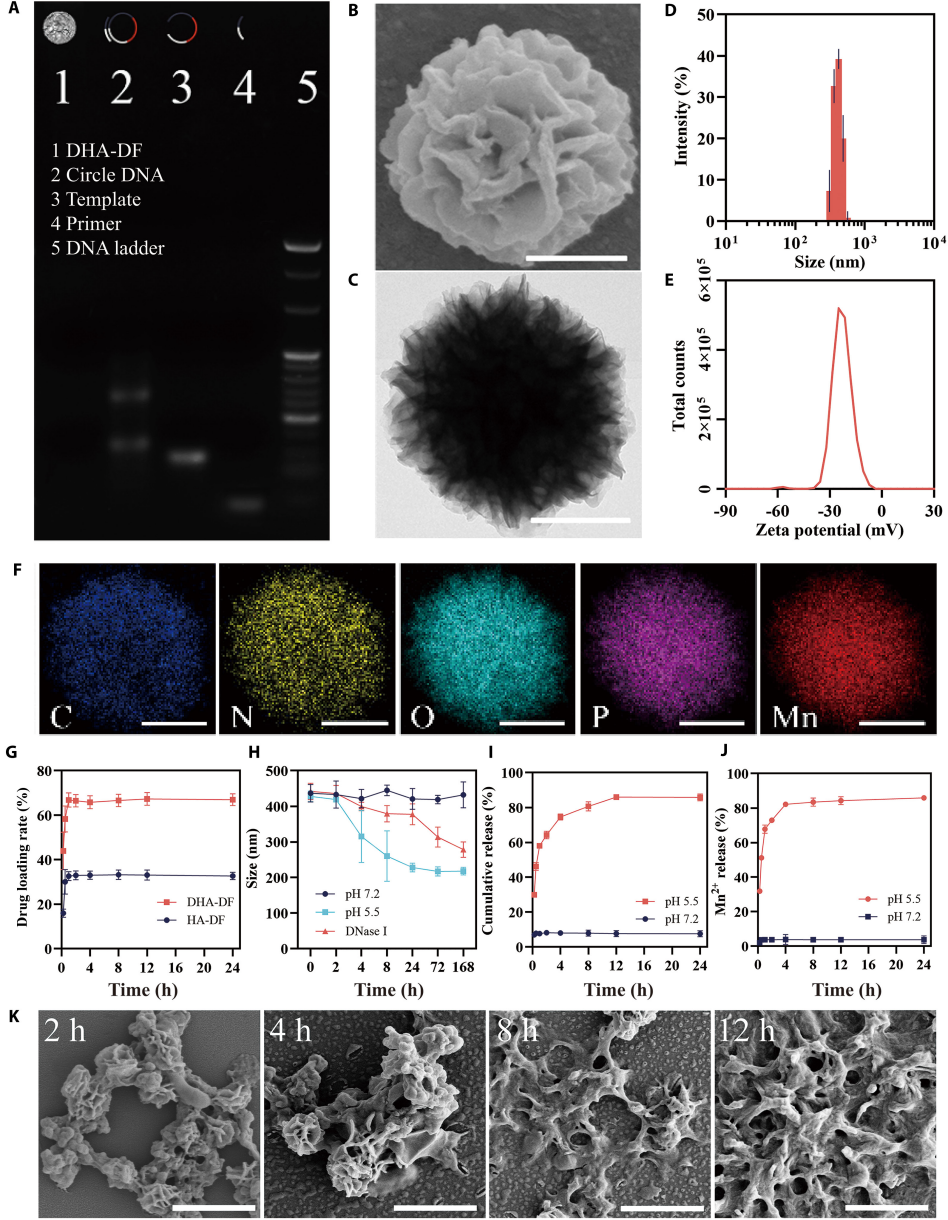
Characterization of DNA nanoflowers. (A) Agarose gel analysis of synthesized DHA-DF. (B) SEM image and (C) TEM image of DHA-DF. Scale bar, 200 nm. (D) Average size and (E) zeta potential of DHA-DF. (F) Elemental mapping of DHA-DF. Scale bar, 200 nm. (G) Time-efficiency diagram of DFs loading DOX. (H) Average size of DHA-DDF under various conditions. (I) Time-efficiency diagram of DHA-DDFs releasing DOX. (J) Time-efficiency diagram of DHA-DDFs releasing Mn^2+^. (K) SEM images of DHA-DDF treated with MES (pH 5.5) for 2, 4, 8, and 12 h. Scale bar, 1 μm.

We have created a DNA template (DHA) with a DOX-loading sequence (D), a sequence encoding the HIF-1α antisense sequence (H), and a sequence encoding the AS1411 aptamer sequence (A). Using a process called rolling cycle amplification (RCA), cDNA, which is produced by cyclizing the linear DNA template, was utilized to make numerous copies of a DNA fragment in order to synthesize DF. Through the RCA procedure, DHA-DF was created from the DHA template (Table S1) in this study. Other nanoflowers, like HA-DF (lacking the HIF-1α ASO sequence), using their corresponding DNA template (Table S1), was also synthesized through RCA. Figure S1 displayed the predicted secondary structures of the pentamer RCA products and the linear DHA template using the Nupack software with a parameter set at 37 °C. The RCA products were analyzed by gel electrophoresis, SEM, and TEM (Fig. 2A to C).

Our design states that the stable structure and DOX-loading site of DHA-DF will effectively load DOX under neutral physiological conditions, minimizing its deleterious effects on normal cells. Drug delivery to cancer cells targeted by DHA-DF would be made possible by the AS1411 aptamer. When DHA-DF comes into contact with the acidic environment surrounding cancer cells, its structure disassembles, releasing the therapeutic components and improving efficacy while reducing off-target damage. SDT is more effective when DOX functions as a sensitizer and HIF-1α ASOs in DHA-DF may lower the expression of HIF-1α of tumor cells.

### Physical and chemical characterization of DF

The molecular weights of RCA products were detected by 2% agarose electrophoresis. As shown in Fig. 2A, there were 2 bands in lane 2, with the cyclization product exhibiting a larger molecular weight than a linear DNA template (lane 3), indicating the formation of cDNA. RCA products remained at the sample well (lane 1), indicating that they had a huge molecular weight. SEM and TEM analyses confirmed that the RCA product exhibited the typical porous petal-like structure observed in these microscopy techniques (Fig. 2B and C). The average size (407.9 ± 13.6 nm, *N* = 5) and zeta potential (−22.4 ± 2.5 mV, *N* = 5) of DFs were measured by dynamic light scattering (DLS), as shown in Fig. 2D and E. By controlling the reaction duration of RCA, DFs of different sizes could be obtained (Fig. S2). The larger the size of the DF was, the more complex its structure was. The EDS elemental mapping (Fig. 2F and Fig. S3) and x-ray photoelectron spectroscopy spectra analysis (Fig. S4) showed that C, N, O, P, and Mn are main elements of DHA-DF.

### Drug loading, stability, and pH responsibility of DF

DHA-DDF is obtained by mixing DHA-DF with DOX drug. Upon loading DHA-DF with DOX, it results in the formation of a red precipitate, which is clearly visible in the test tube, as shown in Fig. S5. The UV–vis spectra of DHA-DDF were similar to those of free DOX (exhibiting absorption peaks at 233 and 480 nm), whereas DHA-DF had no DOX-specific absorption peak in the region (Fig. S6). The drug loading amount is calculated according to standard curve of DOX (Fig. S7) to determine the drug loading rate. As shown in Fig. 2G and Table S3, the drug loading rate of DHA-DF (67.28 ± 3.19%, *N* = 3) is much higher than that of HA-DF (32.7 ± 1.7%, *N* = 3). This difference can be attributed to the way that DOX binds to DNA. DOX is connected to DNA through hydrogen bonds, and CG base pairs can provide more binding sites than AT base pairs. Since DHA-DF contains a higher proportion of CG base pairs compared to HA-DF, it can therefore load a greater amount of the drug.

The stability in physiological condition and pH responsibility of DHA-DDF were thoroughly evaluated. The particle sizes of DHA-DDF in different conditions were detected by DLS. As shown in Fig. 2H, the size of DHA-DDF in acidic condition (pH 5.5) decreased rapidly in 8 h, the size of DHA-DDF in DMEM culture medium (10% FBS and 5 U/ml DNase I) decreased slowly, and the size of DHA-DDF in normal condition (pH 7.2) remained almost unchanged. The results of 2% agarose gel electrophoresis (Figs. S8 and S9) were similar to the results of DLS analysis, except that the molecular weight of DHA-DDF in acidic conditions remained almost unaffected. The SEM images (Fig. S10) demonstrated that DHA-DDF treated with DMEM decomposed gradually in 7 d. Additionally, the structural changes observed under acidic conditions (pH 5.5) indicated a time-dependent disintegration of the flower-like morphology (Fig. 2K). The physical characteristics and structural integrity of DHA-DDF exhibit a dynamic response to acidic condition, indicating that it has the potential to be used in biomedical fields in pH-responsive applications, such as cancer therapy.

Then, the efficiency of DOX releasing was detected by UV–vis spectrophotometry (Fig. 2I), and the efficiency of Mn^2+^ releasing was detected by ICP-OES (Fig. 2J). The release rate of DOX and Mn^2+^ rapidly increased within the first 8 h in acidic conditions (pH 5.5) and then stabilized. In an acidic condition, the hydrogen bond was activated to release DOX, and the Mn_2_PPi was decomposed to release Mn^2+^.

These features mentioned above improve drug delivery efficiency and prevent DHA-DDF from releasing drugs in advance prior to reaching the tumor site. Unlike normal tissues, the tumor microenvironment is characterized by its acidity and hypoxia conditions to which DHA-DDF can quickly respond and release drugs upon arrival in the tumor microenvironment.

### US-mediated DHA-DDF enhanced ROS storm via Mn^2+^/DOX synergy

**Fig. 3.**
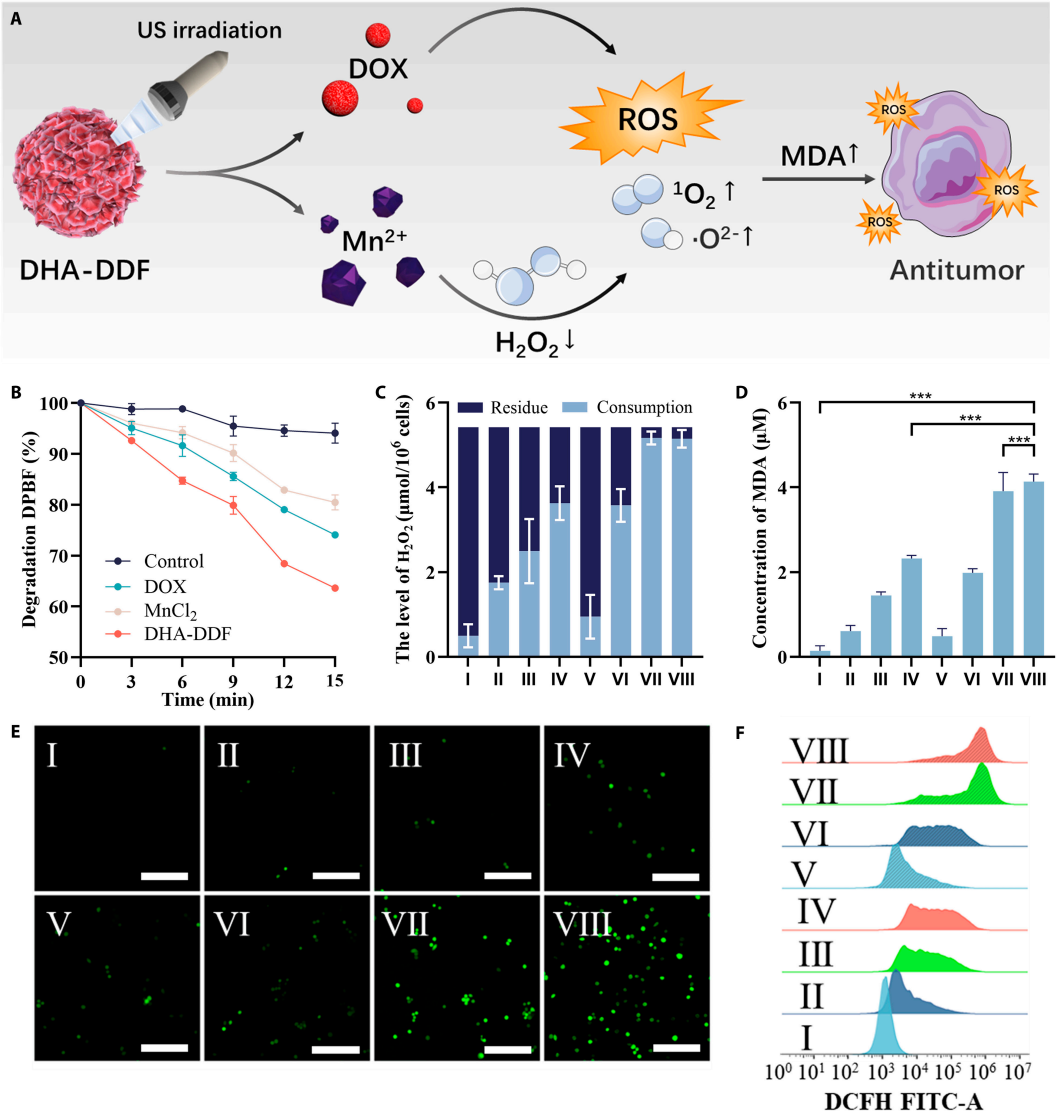
US-mediated DHA-DDFs amplify ROS generation in vitro. (A) Schematic diagram of ROS generation mechanism of DHA-DDF under US irradiation. (B) Consumption of DPBF after various treatments. (C) Residue and consumption of H_2_O_2_ after various treatments. (D) Concentration of MDA after various treatments. (E) CLSM images of singlet oxygen (^1^O_2_) after various treatments. Scale bar, 100 μm. (F) Flow cytometric analysis of ^1^O_2_ generation after various treatments. I: PBS, II: DOX, III: DA-DDF, IV: DHA-DDF, V: PBS + US, VI: DOX + US, VII: DA-DDF + US, VIII: DHA-DDF + US. ***P < 0.001.

Figure 3A schematically illustrates the therapeutic mechanism of DHA-DDF under US irradiation. The Mn^2+^-based nanoflowers selectively target tumor-overexpressed H₂O₂, catalytically converting it into cytotoxic ROS. Meanwhile, the incorporated DOX acts as a sonosensitizer, further enhancing ROS generation upon US exposure, destroying the cellular antioxidant defense system, elevating the level of malondialdehyde (MDA), resulting in severe oxidative stress, and ultimately inducing tumor cell death. To evaluate the ROS-generating capacity of DHA-DDF, we employed DPBF, a chemical probe that specifically degrades upon reaction with singlet oxygen (^1^O_2_​). Strikingly, the DHA-DDF group exhibited the most rapid decrease in DPBF, producing 1.40- and 1.86-fold higher ^1^O_2_​ level compared to the DOX and MnCl₂ groups (Fig. 3B). While DOX intrinsically generates ROS through DNA intercalation and mitochondrial disruption, our DHA-DDF platform synergistically amplifies this effect via Mn^2+^ catalyzed reactions, demonstrating the role of Mn^2+^ in catalyzing ROS generation under US stimulation, likely through a Fenton-like reaction mechanism where Mn^2+^ converts with tumor-endogenous H₂O₂ in tumor into highly cytotoxic ^1^O_2_ and ·O^2−^ [33–36]. Supporting this mechanism, H₂O₂ consumption assay revealed that the DHA-DDF + US group achieved near-complete depletion of H₂O₂ in LLC cells (Fig. 3C), with 5.04% residual H₂O₂ remaining compared to 67.70% in the DOX group. Further validation came from MDA assays, a marker of lipid peroxidation induced by ROS [34]. DHA-DDF + US-treated LLC cells showed the highest MDA levels (6.80-fold higher than the DOX group; Fig. 3D), confirming that Mn^2+^-driven ROS production led to pronounced oxidative damage.

Next, we performed confocal microscopy using SOSG (a ^1^O_2_-specific fluorescent probe). Strong green fluorescence was observed in DHA-DDF + US-treated LLC cells (Fig. 3E), validating the DPBF results. Flow cytometry with SOSG and DHE (for ·O^2−^) further quantified ROS levels, demonstrating that DHA-DDF + US generated higher ^1^O_2_ and ·O^2−^ than free DOX (Fig. 3F and Fig. S11). The minor ROS induction by DOX alone highlights the nanoflower’s advantage: Under US irradiation, Mn^2+^ not only augments DOX’s inherent ROS production but also establishes a self-sustaining cycle to convert tumor-endogenous H₂O₂ into cytotoxic ^1^O_2_ and ·O^2−^. This dual action creates an oxidative storm that overwhelms cellular antioxidant defenses, ultimately triggering tumor cell death.

### Nucleolin-targeted delivery and lysosomal trafficking of DHA-DDF

**Fig. 4.**
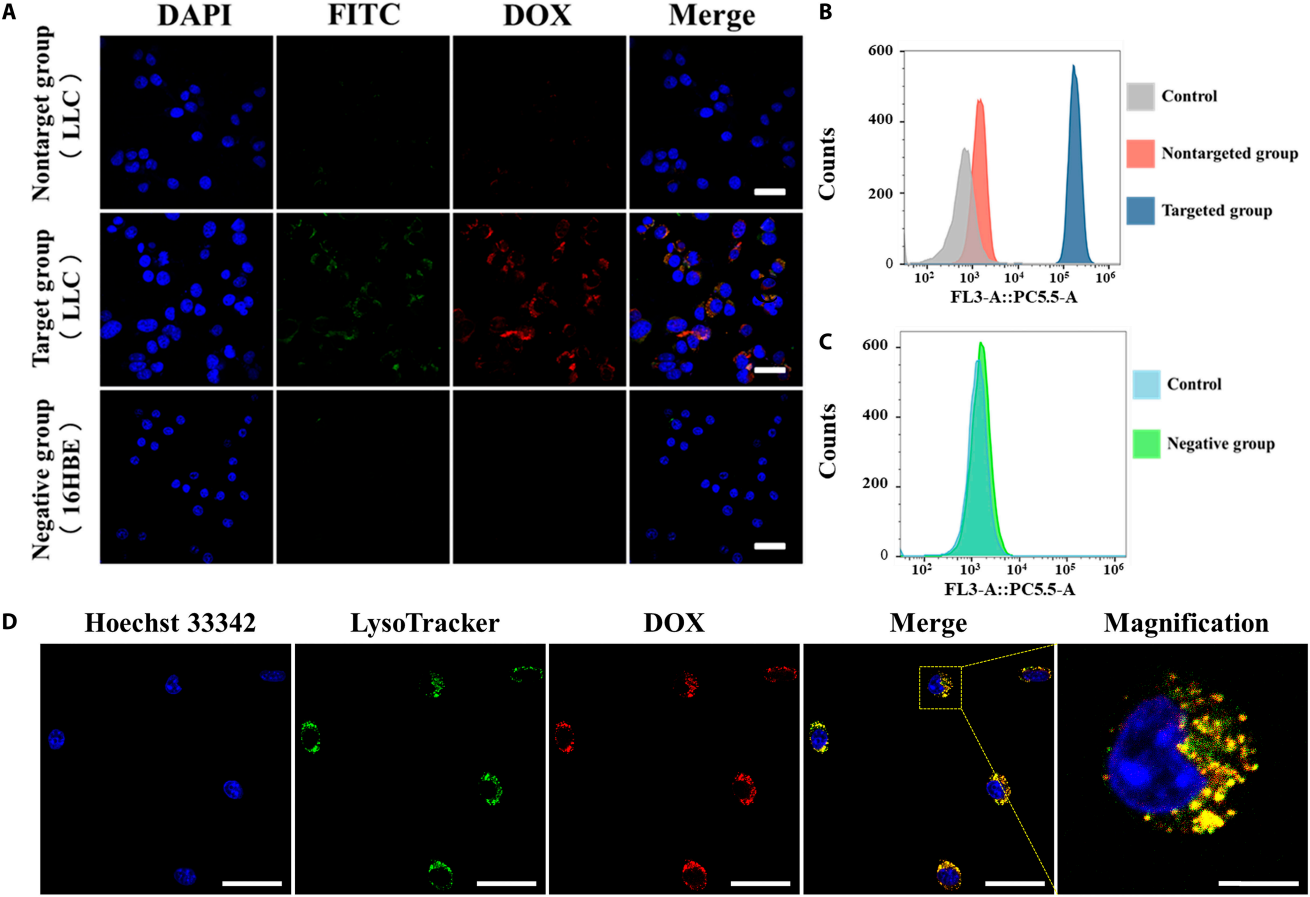
Targeting capacity of DHA-DDF in vitro. (A) CLSM images and (B and C) flow cytometric analysis of colocalization (Nontarget group: FITC-labeling DH-DDF with LLC cells; Target group: FITC-labeling DHA-DDF with LLC cells; Negative group: FITC-labeling DHA-DDF with 16HBE cells). Scale bar, 30 μm. (D) CLSM images of lysosome colocalization of DHA-DDF. Scale bar, 50 μm; inset scale bars, 10 μm.

**Fig. 5.**
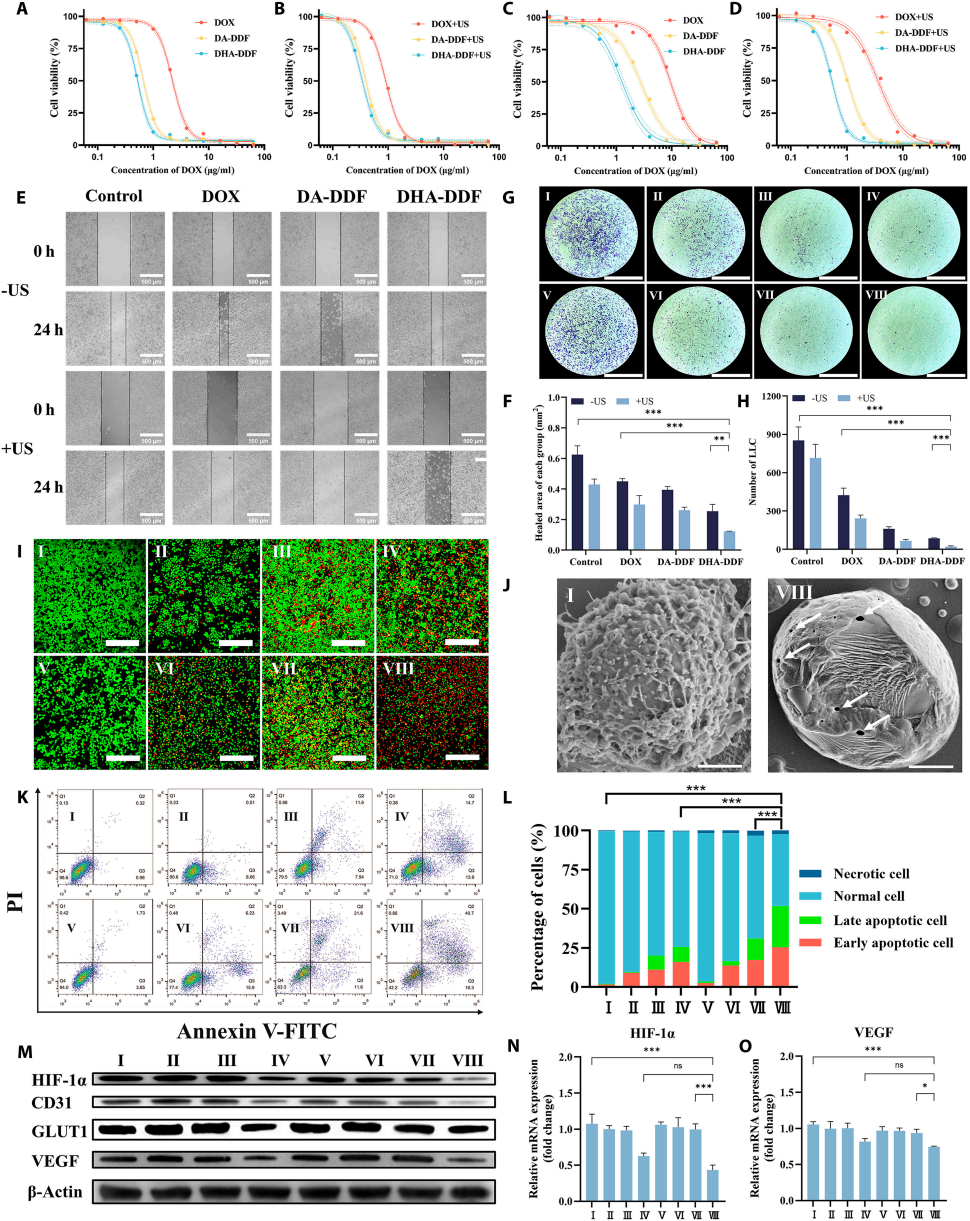
DHA-DDF enhances the efficacy of SDT in vitro. (A to D) Cell viability of LLC cells treated with DOX, DA-DDF, or DHA-DDF. (A) Without US irradiation under normoxic condition. (B) With US irradiation under normoxic condition. (C) Without US irradiation under hypoxic condition. (D) With US irradiation under hypoxic condition. (E) Representative images of wound healing assay and (F) quantitative analysis of cell migration area following various treatments. Scale bar, 500 μm. (G) Representative images of transwell assay and (H) quantitative analysis of invaded cells following various treatments. Scale bar, 500 μm. (I) CLSM images of live cells (green) and dead cells (red) stained following various treatments. Scale bar, 200 μm. (J) SEM images of LLC cells in group I (left) and group VIII (right). Scale bar, 2 μm. (K) Flow cytometry analysis and (L) quantitative apoptosis proportion plot following various treatments. (M) Western blot analysis of hypoxia- and angiogenesis-related proteins expression. (N) RT-qPCR analysis of hypoxia-related gene and (O) angiogenesis-related gene. I: PBS, II: DOX, III: DA-DDF, IV: DHA-DDF, V: PBS + US, VI: DOX + US, VII: DA-DDF + US, VIII: DHA-DDF + US. *P < 0.05, **P < 0.01, ***P < 0.001.

The AS1411 aptamer exhibits specific binding affinity for nucleolin, a transmembrane protein overexpressed in cancer cells [37,38]. By incorporating the AS1411 aptamer sequence into DF, we engineered DHA-DDF with precise tumor-targeting capability, demonstrating higher fluorescence intensity in nucleolin-high LLC cells compared to nucleolin-low 16HBE normal lung epithelial cells by qualitative observation through confocal microscopy and quantitative analysis through flow cytometry (Fig. 4A to C). This selective binding translated to potent tumor-specific cytotoxicity, with DHA-DDF showing 91.92% LLC cell death versus 7.80% in 16HBE cells at equivalent doses (2 μg/ml and 24 h; Fig. 5A and Fig. S12), a remarkable advantage over conventional DOX that simultaneously attacks both malignant and healthy tissues. The limited use of DOX in clinical therapy is due to its potent toxic side effects, including DOX-induced cardiotoxicity [39]. Fortunately, the selective targeting of DHA-DDF, which is loaded with DOX, toward cancer cells with high nucleolin expression limits its impact on normal lung epithelial cells.

Encouraged by the targeting capacity of DHA-DDF, we next analyzed the colocalization between DHA-DDF and the lysosome of LLC cells. The results indicated that DHA-DDF binds to nucleolin on the cell membrane as shown in Fig. 4A. Further analysis revealed that DHA-DDF subsequently enters the cell and colocalizes with lysosomes (Fig. 4D). These findings suggest that DHA-DDF is likely to first bind to nucleolin on the cell membrane and then enters the cell via the lysosomal endocytosis pathway. This pathway not only enhances tumor specificity but also explains the reduced systemic toxicity, which the lysosomal barrier prevents sudden DOX bursts that damage cardiomyocytes, while allowing gradual drug release in cancer cells after lysosomal escape, demonstrating DHA-DDF’s clinical potential for overcoming DOX’s cardiotoxicity challenge.

### HIF-1α ASO overcomes hypoxia-induced resistance and boosts SDT

IC_50_ (median inhibitory concentration) values for DOX and DHA-DDF in LLC cells in normoxic conditions and hypoxia conditions were determined (Fig. 5A to D). It was observed that the IC_50_ of DOX in LLC cells under hypoxic condition stood at 9.71 μg/ml, which was higher than the IC_50_ under normoxic conditions at 2.22 μg/ml (Table S4). This discrepancy highlights the drug resistance induced by hypoxia in cancer cells. Regarding DHA-DDF, CCK-8 cytotoxicity assays confirmed the superior safety profile of DHA-DDF compared to free DOX (Fig. S12). Using 16HBE (normal human bronchial epithelial cells) as a model, we observed obviously higher cell viability in DHA-DDF-treated groups compared to DOX-treated groups at equivalent concentrations, demonstrating the reduced toxicity of this nanoplatform in normal cells. Furthermore, DHA-DDF incorporates the sequence encoding the HIF-1α ASOs; its IC_50_ (1.28 μg/ml) in LLC cells was found to be lower than that of DA-DDF (lacking the HIF-1α ASO sequence, 2.87 μg/ml) under hypoxic conditions, especially under ultrasonic stimulation (0.54 and 0.98 μg/ml, respectively). However, this difference was not obviously under normoxic conditions. The differential efficacy likely stems from the HIF-1α ASO in DHA-DDF, which selectively enhances cytotoxicity under hypoxia by inhibiting HIF-1α-driven survival pathways. As HIF-1α is known to accumulate and orchestrate cellular adaptation to low oxygen conditions, its targeted inhibition by the ASO potentiates drug efficacy. In contrast, under normoxic conditions, HIF-1α undergoes rapid ubiquitin-proteasomal degradation, rendering the ASO component ineffective.

The antisense technique is a means of interfering with gene expression, and the most commonly used ASOs are also considered novel therapeutic agents [40]. Compared with traditional drugs, ASO binds to the targeted RNA through Watson–Crick base pairing, which makes higher efficiency and less drug resistance [41,42]. With the advantages of high specificity, easy design diversity, and simple synthesis, ASOs have been used to treat various diseases [41]. By the inclusion of HIF-1α ASO into DHA-DDF, we can effectively target and suppress this critical regulator of the hypoxic response, thereby enhancing therapeutic effectiveness.

So far, more than 100 kinds of HIF-1α-related target genes have been identified, including those involved in angiogenesis, glucose transport, and glycolysis [43]. HIF-1α up-regulation is known to promote processes such as tumor proliferation, invasion, metastasis, and immune escape under hypoxia conditions [44]. Meanwhile, HIF-1α induced multidrug resistance in tumor cells by up-regulating the expression of efflux drug transporters in hypoxia [45]. Western blotting analysis demonstrated a decrease in the level of HIF-1α, CD31, GLUT1, and VEGF protein in LLC cells after treatment with DHA-DDF or DHA-DDF + US (Fig. 5M). Meanwhile, real-time qPCR (RT-qPCR) analysis demonstrated that the combination of DHA-DDF and US markedly down-regulated the gene expression of HIF-1α and VEGF (Fig. 5N and O and Table S5), suggesting a potential mechanism for hypoxia and angiogenesis modulation. Specifically, by reducing HIF-1α levels, HIF-1α ASO could sensitize tumor cells to therapy and enhance the efficacy of SDT. This synergistic approach offers a strategic solution to overcome hypoxia-induced treatment resistance and improve SDT outcomes.

### Optimized US parameters and combinatorial therapy

Subsequently, we optimized the US parameters (Fig. S13) and identified 1 W/cm^2^ for 3 min as the optimal condition for LLC cell treatment. This intensity and duration provided effective cytotoxicity without greatly reducing cell viability (>80% survival rate) in the absence of DA-DDF or DHA-DDF, ensuring that the observed therapeutic effects in subsequent combinatorial experiments were primarily attributable to the synergistic action of US and nanoflowers. In the following experiments, 8 different regimens were used to treat LLC cells: I (PBS), II (DOX), III (DA-DDF), IV (DHA-DDF), V (PBS+US), VI (DOX + US), VII (DA-DDF + US), and VIII (DHA-DDF + US). The cells were incubated with culture medium containing PBS, DOX, DA-DDFs, or DHA-DDFs (equal to 2 μg/ml DOX) for 4 h. After washing with PBS, the cells in the US-related groups were exposed to the ultrasonic radiation in the dark. As shown in Fig. 5K and L, there was the highest level of apoptosis induced in LLC cells from group VIII, which received treatment with DHA-DDF in conjunction with SDT. The pattern was also supported by the results of the wound healing assay (Fig. 5E and F) and transwell assay (Fig. 5G and H). Furthermore, cells from group VIII were observed under SEM, finding that they have lost their cell membrane on the cell surface, and tiny pores (white arrows) appeared on the cell wall after treatment (Fig. 5J). These results demonstrated that DHA-DDF augmented by SDT exhibits potent inhibitory effects on the proliferation, migration, and invasion of LLC cells under hypoxia conditions.

### Metabolic reprogramming and tumor microenvironment modulation

Actually, the excessive acidic metabolites in hypoxia conditions such as lactic acid (LA) are in the basement of the tumor acidic microenvironment, which induces immune suppression [46]. Therefore, we measured the levels of glucose and LA in the culture medium of LLC cells after various treatments (Figs. S14 and S15). Compared to the other groups, the glucose consumption and LA production of group VIII decreased obviously. It proved that down-regulating HIF-1α can inhibit LA accumulation and potentially improve the tumor microenvironment for enhanced cancer therapy.

To sum up, by specifically targeting and down-regulating HIF-1α expression, HIF-1α ASO effectively overcomes tumor cell treatment resistance while substantially enhancing the treatment efficacy of SDT. This strategy offers a crucial advantage in overcoming hypoxia-induced treatment resistance, thereby substantially improving SDT therapeutic outcomes.

### In vivo drug target, distribution, and release of DHA-DDF

**Fig. 6.**
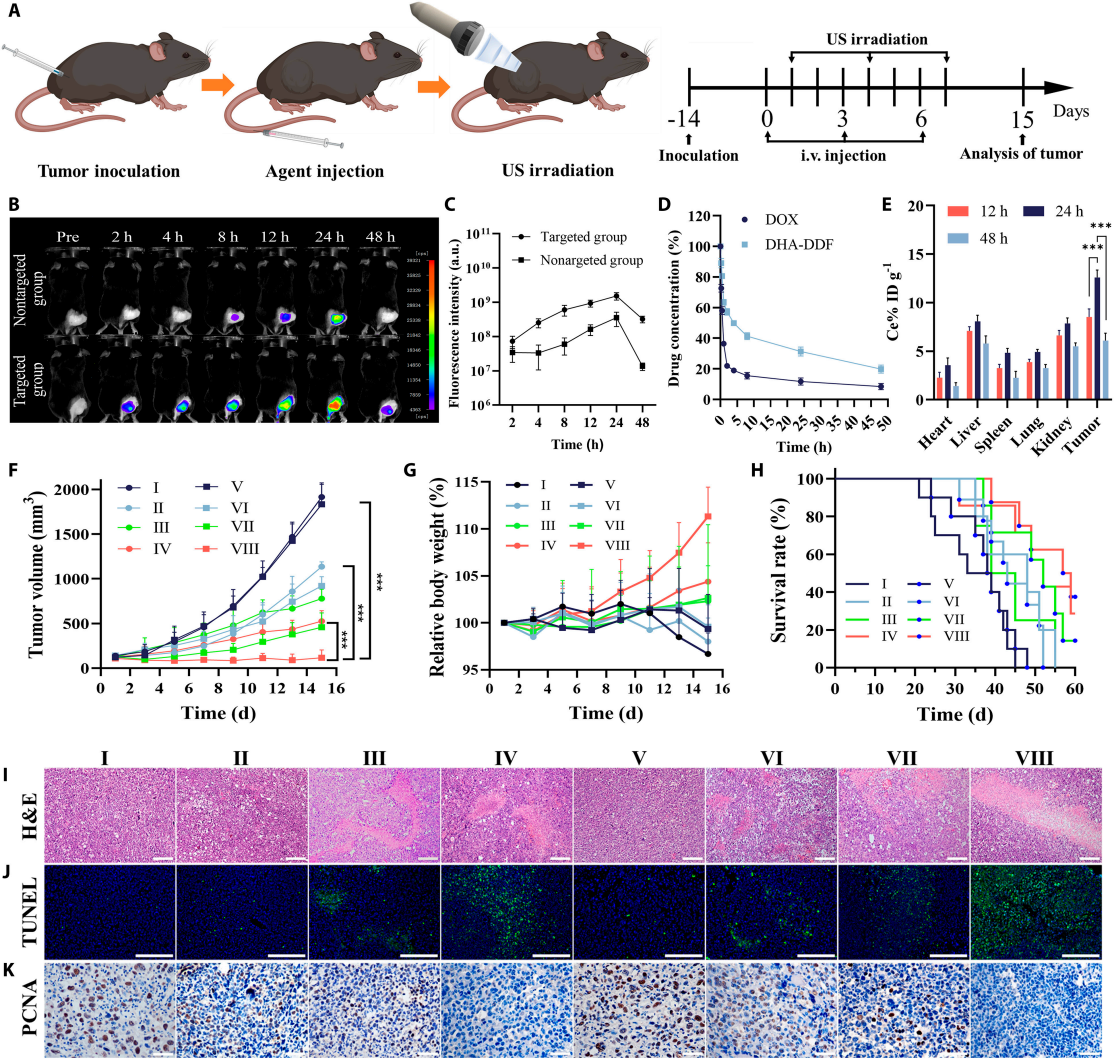
Evaluation of drug distribution, pharmacokinetics, and antitumor effect of DHA-DDF in vivo*.* (A) Schematic diagram of treatment plan. (B) In vivo fluorescence images and (C) quantitative fluorescence intensity of tumor-bearing mice at different times after injecting Cy5-DH-DDF (nontargeted group) and Cy5-DHA-DDF (targeted group). (D) DOX content in tumors and major tissues of LLC-bearing mice following injection of DHA-DDF. (E) In vivo pharmacokinetics in female Sprague–Dawley rats after injection of DOX and DHA-DDF. (F) Tumor volume, (G) relative body weight, and (H) survival rate of each group after 15 d of different treatments. (I) Representative images of H&E staining sections from tumors of different groups. Scale bar, 200 μm. (J) Representative images of TUNEL fluorescence staining sections from tumors of different groups. Scale bar, 200 μm. (K) Representative images of PCNA staining sections from tumors of different groups. Scale bar, 50 μm. I: PBS, II: DOX, III: DA-DDF, IV: DHA-DDF, V: PBS + US, VI: DOX + US, VII: DA-DDF + US, VIII: DHA-DDF + US. ***P < 0.001.

To systematically evaluate the tumor-targeting properties and biodistribution profile of DHA-DDF, we performed longitudinal fluorescence imaging for 48 h following intravenous administration of Cy5-labeled DHA-DDF and Cy5-labeled DH-DDF in tumor-bearing mice. As shown in Fig. 6B, time-course imaging revealed initial tumor accumulation within 2 h post-injection, with signal intensity progressively increasing to peak at 24 h before declining by 48 h. Quantitative analysis demonstrated 23.77-fold fluorescence intensity in the targeted group (Cy5-DHA-DDF) compared to the nontargeted group (Cy5-DH-DDF) at 48 h (Fig. 6C). To further investigate the biodistribution pattern, we conducted ex vivo fluorescence imaging of major organs (heart, liver, spleen, lung, kidney) and tumor tissues at the 48-h endpoint (Fig. S16). The results showed pronounced fluorescence signals in both tumor tissues and the hepatorenal system (liver and kidney), while other organs exhibited minimal fluorescence. This distinct distribution pattern suggests that while DHA-DDF effectively targets tumor tissue, it might undergo primary elimination through hepatic and renal clearance pathways.

To further investigate the pharmacokinetic behavior of DHA-DDF, we systematically monitored blood drug concentrations following intravenous administration. As shown in Fig. 6D, pharmacokinetic analysis revealed distinct profiles, where free DOX exhibited rapid clearance from circulation, whereas DHA-DDF demonstrated considerably enhanced plasma retention, as evidenced by a greater area under the concentration–time curve and prolonged elimination half-life compared to free DOX. These findings substantiate that DHA-DDF effectively extends the systemic circulation time and improves the bioavailability of DOX, thereby promoting its tumor-selective accumulation via the enhanced electron paramagnetic resonance (EPR) effect.

Complementary tissue distribution studies were performed using UV–vis spectroscopy to quantify DOX levels in tumor tissues and major organs of LLC-bearing mice at various time points (Fig. 6E). Specifically, DHA-DDF exhibited exceptional tumor-targeting capability, with peak accumulation at 24 h post-injection (12.6% ID g^−1^) and sustained retention at 48 h (6.1% ID g^−1^), demonstrating active tumor-selective deposition through both EPR effects and AS1411-mediated targeting. Remarkably, the minimal cardiac accumulation (<5% ID g^−1^ at all time points) contrasts sharply with the severe cardiotoxicity of free DOX. Besides, consistent with classical nanoparticle clearance pathways, accumulation in liver and kidneys (peaking at 24 h followed by 48-h decline) suggests hepatobiliary and renal clearance pathways, which aligns with the fluorescence distribution pattern in ex vivo imaging (Fig. S16). This clearance pattern is particularly advantageous for clinical translation, as hepatorenal elimination is a well-characterized pathway for U.S. Food and Drug Administration-approved nanotherapeutics. Most importantly, the consistent 48-h depletion kinetics across all tissues informed our rational dosing regimen (inject once every 48 h; Fig. 6A), ensuring repeated drug delivery while preventing systemic accumulation. These results collectively validate DHA-DDF’s dual advantage: effective tumor targeting coupled with physiological clearance mechanisms that minimize off-target toxicity.

### In vivo antitumor efficacy

Prior to in vivo evaluation, we systematically assessed the biocompatibility of DHA-DDF. Hemolysis assays (Fig. S17) demonstrated that DHA-DDF exhibited lower hemolysis rate compared to free DOX at equivalent concentrations, confirming its superior blood compatibility for systemic administration. To establish safe SDT parameters, we then performed comprehensive biosafety evaluations of US irradiation. H&E staining identified noticeable dermal tissue damage in murine gluteal regions when exposed to US at 1.9 W/cm^2^ for 5 min (Fig. S18). This critical finding defined the upper safety limit for US intensity and duration, providing essential guidance for the combined use of DHA-DDF with SDT while maintaining tissue integrity.

Encouraged by its remarkable tumor-targeting capability, high tissue accumulation, and favorable safety profile, we further investigated the antitumor efficacy of DHA-DDF in LLC-bearing mice. Mice were administered PBS, free DOX, DA-DDF, or DHA-DDF on days 0, 3, and 6. In the US irradiation groups, tumors were exposed to US (1.0 W/cm^2^, 3 min) 24 h after each injection (Fig. 6A). Tumor volume and body weight were monitored over 15 d, while survival rates were tracked for 60 d. Notably, mice treated with DHA-DDF with US irradiation exhibited the most pronounced suppression of tumor growth (Fig. 6F and Fig. S19). As tumors progressed, mice in the PBS group showed a notable decline in body weight (Fig. 6G), whereas the DHA-DDF + US group demonstrated the lowest tumor burden and the highest survival rate throughout the 60-d observation period (Fig. 6H). Meanwhile, the necrotic areas were observed on the paraffin section of the tumor tissue by H&E staining, and apoptotic regions were observed on the frozen section of the tumor tissue by TUNEL staining. As shown in Fig. 6I and J, no apoptotic or necrotic areas were found in the tissue sections of group I, but group VIII had the largest area of these phenomena. It proved that DHA-DDF with US could effectively promote tumor tissue apoptosis and necrosis.

PCNA, an evolutionarily conserved protein in all eukaryotic cells, is widely used as a marker for tumor progression, plays an essential role in regulating DNA synthesis and repair, and is indispensable to cancer cell growth and survival [47]. To further evaluate the efficacy, we investigated the expression of PCNA by immunohistochemical staining. As depicted in Fig. 6K, the tumor tissue in groups treated with free DOX, DA-DDF, or their combination with US irradiation exhibited high levels of PCNA with minimal inhibitory effects on tumor growth. In contrast, DHA-DDF effectively reduced PCNA levels, and when combined with US irradiation, it showed even more distinct inhibitory effects. These findings highlight the therapeutic potential of DHA-DDF. By integrating tumor targeting, controlled drug release, and SDT enhancement, DHA-DDF achieves potent in vivo antitumor effects, offering a viable clinical approach for tumor treatment.

### In vivo tumor hypoxia alleviation and vascular normalization

**Fig. 7.**
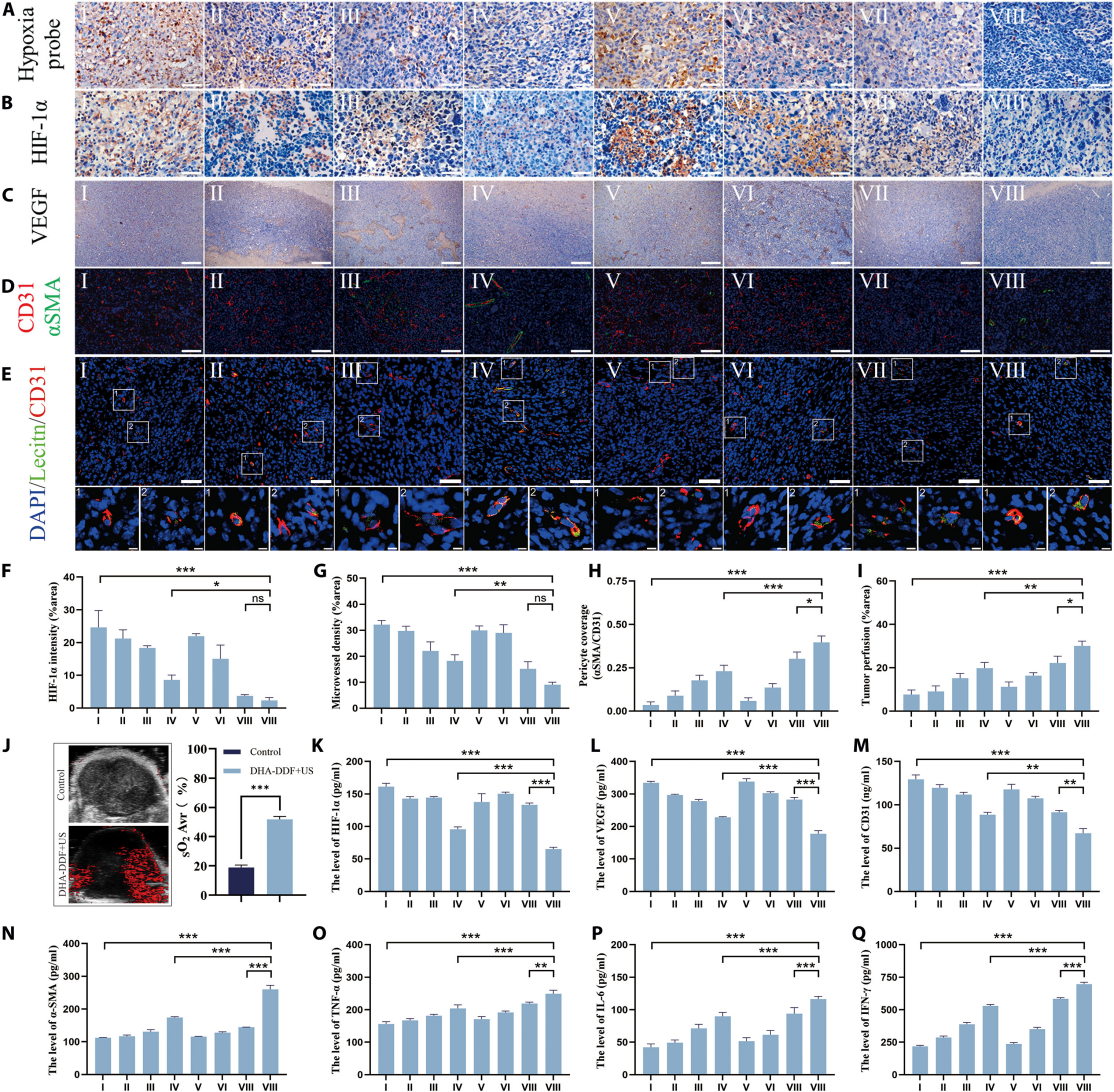
The synergistic effect of US-mediated DHA-DDF on the level of hypoxia-related factors and vascular normalization in tumor tissues and serum. (A) Immunohistochemical images of pimonidazole staining as a marker of hypoxia in tumor sections from mice after various treatments. Scale bar, 50 μm. (B) Immunohistochemical staining of HIF-1𝛼 in tumor sections from mice after various treatments. Scale bar, 50 μm. (C) Immunohistochemical staining of VEGF in tumor sections from mice after various treatments. Scale bar, 50 μm. (D) Immunostaining of tumor vessels covered by pericytes from mice after various treatments. Scale bar, 50 μm. (E) Immunostaining of tumor vessel perfusion from mice after various treatments. Scale bar, 50 μm. (F) Quantification of HIF-1𝛼 intensity based on the images from (B). (G and H) Quantification of microvessel density and pericyte-covered tumor vessels based on the images from (D). Microvessel density was determined by calculating the CD31-positive area/tumor area in fluorescence images. Pericyte coverage was determined by calculating the α-SMA-positive area/CD31-positive area in fluorescence images. (I) Quantification of tumor vessel perfusion based on the images from (E). Tumor vessel perfusion was determined by calculating the lectin-positive area/CD31-positive area in fluorescence images. (J) Representative in vivo PA images of sO₂ and levels within the tumor tissue before and after treatment. (K to Q) ELISA analysis of cytokine levels in mouse serum after various treatments. I: PBS; II: DOX: III: DA-DDF; IV: DHA-DDF; V: PBS + US; VI: DOX + US; VII: DA-DDF + US; and VIII: DHA-DDF + US. *P < 0.05, **P < 0.01, ***P < 0.001.

Given that the ability of HIF-1α ASO cannot produce oxygen directly but might improve tumor blood supply, we investigated the effects of DHA-DDF on hypoxia alleviation in LLC tumor-bearing mice. Tumor hypoxia was assessed using pimonidazole, a nitroimidazole-based hypoxia probe that forms stable adducts in oxygen-deficient tissues. Immunohistochemical analysis demonstrated that DHA-DDF + US substantially alleviate tumor hypoxia, as evidenced by reduced pimonidazole staining and decreased HIF-1α expression (Fig. 7A and B), potentially through vascular normalization-mediated improvement in oxygen perfusion. Quantitative analysis confirmed this therapeutic effect, showing a progressive decrease in HIF-1α expression levels across treatment groups, highest in PBS controls (24.65% positive area), moderate in DHA-DDF alone (8.57% positive area), and minimal in the DHA-DDF + US group (2.32% positive area) (Fig. 7F). Consistent with these findings, PA imaging system revealed a remarkable 2.7-fold increase in tumor tissue oxygen saturation (sO₂), from 18.96% before treatment to 51.81% afterward (Fig. 7J).

The aberrant vasculature structure of tumor, marked by abnormalities and functional impairment, notably exacerbates tumor hypoxia and impedes drug penetration [48]. Thus, vessel normalization, a phenomenon discovered during antiangiogenic therapy, provides a new strategy for tumor therapies [49]. VEGF, a critical factor of vascular vascularization regulated by HIF-1α under hypoxia conditions, can exacerbate vascular malformations by up-regulating CD31 and down-regulating α-SMA expression [50]. To assess the vascular normalization potential of DHA-DDF + US in vivo, we conducted a comprehensive evaluation of tumor vascular structure and function. Immunohistochemical analysis of VEGF revealed that, compared to PBS-treated control, DHA-DDF + US treatment considerably attenuated tumor angiogenesis (Fig. 7C). Quantitative assessment of CD31 immunostaining revealed a 3.56% decrease in CD31-positive microvessel density following DHA-DDF + US treatment (Fig. 7D and G), indicating substantial microvessel remodeling. Concurrently, α-SMA immunostaining showed an 11.58% increase in pericyte coverage (Fig. 7H), suggesting enhanced vessel maturation and stability. Additionally, by using immunofluorescence staining with FITC-labeled lectin, we further evaluated changes in vascular perfusion of tumors, revealing an 3.92% increase following DHA-DDF + US treatment (Fig. 7E and I). The ELISA results further provided quantitative validation of these findings, with reduction in serum levels of HIF-1α, VEGF, and CD31, along with a marked increase in α-SMA expression (Fig. 7K to N). These changes correlated with progressive vascular normalization, leading to substantial alleviation of tumor hypoxia.

To further verify the activation of effector cells, we investigated whether DHA-DDF + US could promote the secretion of pro-inflammatory cytokines, such as TNF-α, IL-6, and IFN-γ, which are important markers in cellular immunity. As shown in Fig. 7O to Q, ELISA results demonstrated that TNF-α, IL-6, and IFN-γ were notably increased in the DHA-DDF + US group (1.59-, 2.76-, and 3.19-fold) compared to those in the control group. From these data, we concluded that DHA-DDF + US can markedly facilitate the infiltration and activation of immune cells, validating compelling evidence for the activation of antitumor immune responses following combination therapy.

Taken together, these findings demonstrate that DHA-DDF + US promotes potent hypoxia alleviation and vascular normalization through coordinated regulation in angiogenic factor expression, microvessel density, pericyte recruitment, tumor perfusion, and antitumor immunity.

### In vivo biosafety investigation

To evaluate the biosafety of DHA-DDF in vivo, we conducted a comprehensive biosafety assessment following various treatments. Serial serum biochemical analyses were performed at 7, 15, and 30 d post-treatment to evaluate systemic toxicity. As shown in Figs. S20 to S22, hepatic (ALT, AST), renal (BUN, CREA), and muscle (CK) function parameters were within normal reference values, indicating the absence of any disorder or disease condition and confirming the exfig. 7cellent safety profile of DHA-DDF administration. In addition, H&E staining of major organs at 15 and 30 d post-treatment revealed intact tissue architecture across all treatment groups, with no evidence of inflammatory infiltration, congestion, or necrosis, demonstrating that DHA-DDF induces no observable toxicological effects (Figs. S23 and S24). To summarize, DHA-DDF shows strong potential for future clinical translation by being both safe and efficacious in vivo.

## Conclusion

Lung cancer is the most severe deadly disease globally. Low-intensity US triggering used in SDT has shown encouraging anticancer potential. However, the sonodynamic effect on cancer is severely hindered by hypoxia present in tumor tissue. Hypoxia leads to HIF-1α stabilization, which encourages glycolysis in tumor cells and drug resistance.

We designed and synthesized manganese ion-based DNA nanoflowers (DHA-DDF), and when combined with US, could exhibit excellent antitumor effects. The mechanisms include (a) the intrinsic penetrability of low-intensity US, which we confirmed promotes medication into tumor spheroids (Fig. S25); (b) targeted delivery via the AS1411 aptamer sequence in DHA-DDF, enhancing drug efficiency and minimizing DOX toxicity to healthy tissues; (c) pH-responsive release of DOX upon reaching the tumor microenvironment; (d) synergistic ROS generation with DOX and Mn^2+^ under US stimulation, maximizing toxic effects on tumor cells; (e) incorporation of HIF-1α ASO within DHA-DDF to lower HIF-1α levels, sensitizing tumor cells to therapy and reducing IC_50_ of DOX under hypoxic conditions; (f) most importantly, the combined treatment with US normalizes tumor vasculature, facilitating drug delivery to the tumor site and aiding in tumor cell destruction.

This treatment regimen not only reduced tumor cell proliferation and increased tumor cell apoptosis but also alleviated tumor hypoxia and induced vascular normalization. As a result, DHA-DDF combined with US markedly prolonged the lifespan of the mice. This strategy offers an advantageous approach to overcome hypoxia-induced resistance and improve the therapeutic outcomes of SDT.

## Data Availability

All data that support the findings of this study are available from the corresponding author upon reasonable request.
