# Supplementary material for: Sonodynamic Therapy-Based DNA Nanocarriers with Hypoxia-Inducible Factor-1α Silencing Activation for Precision Lung Cancer Therapy
Source: Biomater Res. 2025 Aug 21;29:0230. doi: 10.34133/bmr.0230 (PMC12369946; doi:10.34133/bmr.0230)
Supplement: Supplementary 1 — Figs. S1 to S25 Tables S1 to S5 [file bmr.0230.f1.docx]

**Supplemental Material**

**Sonodynamic Therapy Based DNA nanocarriers with HIF-1α Silencing Activation for Precision Lung Cancer Therapy**

Yuchao Cao^1*^, Shangfeng Shen^1*^, Jiahui Xiang^2^, Yan Qiu^1^，Jiajun Guo^1^，Yuqing Zhang^1^, Dairong Li^2#^ and Yonghong Du^1#^

^1^State Key Laboratory of Ultrasound in Medicine and Engineering, College of Biomedical Engineering, Chongqing Medical University, Chongqing, 400016, China.

^2^Department of Respiratory and Critical Care Medicine, the First Affiliated Hospital of Chongqing Medical University, Chongqing 400016, China.

*These authors contributed equally to this work.

#Co-corresponding author: Dairong Li and Yonghong Du

#Correspondence: Dairong Li

Address: No.1, Youyi Road, Yuzhong District, Chongqing 400016, China.

Tel: +86-23-89012745

Fax: +86-23-68485021

E-mail: lidairong@hospital.cqmu.edu.cn

#Correspondence: Yonghong Du

Address: No.1, Yixueyuan Road, Yuzhong District, Chongqing 400016, China.

Tel: +86-23-68485021

Fax: +86-23-68485021

E-mail: duyonghong@cqmu.edu.cn

**Table S1.** Sequences of DNA oligonucleotides

| Oligonucleotide | Sequences (5’-3’) |
| --- | --- |
| DHA Template | Phosphate-TGCTGCGGCATATCGTACGATATGCCATAGCGATACGCGTATCG  CTGCAGCTTGGACACTGGTGGCTCATTCCACCACCACCAACACCACC  ACCACC |
| DA Template | Phosphate-TGCTGCGGCATATCGTACGATATGCCATAGCGATACGCGTATCG  CTGCAGCAAAAAAAAAAAAAAAAAAAACCACCACCACCAACACCACC  ACCACC |
| DH Template | Phosphate-TGCTGCGGCATATCGTACGATATGCCATAGCGATACGCGTATCG  CTGCAGCTTGGACACTGGTGGCTCATTAAAAAAAAAAAAAAAAAAAAA  AAAAA |
| HA Template | Phosphate-AAAAAAAAAAAAAAAAAAAAAAAAAAAAAAAAAAAAAAAA  AAAAAAAAAAATTGGACACTGGTGGCTCATTCCACCACCACCAACACCACCAC  CACC |
| DHA Primer | TATGCCGCAGCAGGTGGTGGTGGT |
| DH Primer | TATGCCGCAGCATTTTTTTTTTTT |
| HA Primer | TTTTTTTTTTTTGGTGGTGGTGGT |
| Drug Loading | TGCTGCGGCATATCGTACGATATGCCATAGCGATACGCGTATCGCTGCAGC |
| AS1411 aptamer | CCACCACCACCAACACCACCACCACC |
| HIF-1α ASO | TTGGACACTGGTGGCTCATT |

**Table S2.** Calculation of dNTP consumption and replication efficiency of DF assembly for 8 h

|  | DHA-DF | DH-DF | DA-DF | HA-DF |
| --- | --- | --- | --- | --- |
| Total dNTP concentration(mM) | 8 | 8 | 8 | 8 |
| OD260 of Remnant dNTP (10× dilution) | 1.790056 | 3.1236667 | 2.8963333 | 4.5516667 |
| Average extinction coefficient of dNTP | 11950(cm·M)^-1^ | 11950 (cm·M) ^-1^ | 11950 (cm·M) ^-1^ | 11950 (cm·M) ^-1^ |
| Remnant dNTP concentration (mM) | 1.49795 | 2.61394 | 2.42371 | 3.80893 |
| Consumed dNTP (mM) | 6.50205 | 5.38606 | 5.57629 | 4.19107 |
| Average replicated copies (0.5 µM 97-nucleotide template) | 134.062795 | 114.052783 | 114.975052 | 86.413814 |

**Table S3.** Calculation of DOX loading in DHA-DF.

| Total DOX | 90μL,1 mg |
| --- | --- |
| Total volume of DFs | 10μL |
| Total DOX-binding capacity in DFs (mole) | 10 µL × 0.5 µM × (134.1+1) DNA copies per template  × (12 drug-binding sites per copy |
| Total loaded DOX (mole) | (100 µL DF-DOX mixture × 0.0217 mM loaded DOX) |
| %Maximal DOX loading by specific DNA-binding | 100 × (Total DOX-binding capacity in NFs)/ (Total loaded  DOX) =34.54 |
| %DOX loading capacity | 100 × (580 g/mole DOX × Total loaded DOX)/ (29627.19  g/mole template × 0.0005 mM template × (134.1 + 1)  DNA copies per template × 10 µL DFs) =67.28 |

Notes:

1.(134.1+1) total DNA copies per template in final product, including the template (1) and the replicates (134.1).

2.Drug-binding sites: a maximum of 12 tandem GC or CG sites in each copy of DNA replicates or templates.

3.Molecular weight of DOX hydrochloride: 580 g/mole.

4.Molecular weight of one of RCA replicate: 29627.19 g/mol, assuming that the template has a molecular weight similar to that of its replicates.

Equations used:

Total DOX binding capacity = (total DF volume) × (concentration of DNA copies) × (number of DOX binding sites per DNA copy)

Total Loaded DOX = (Volume of DF_DOX complexes) × (DOX concentration in DF_DOX complexes)

% (Maximal DOX loading by specific DNA binding) = 100 × (Total DOX binding capacity in DFs) / (Total loaded DOX)

% (Drug loading capacity) = 100 × (weight of total loaded DOX) / (weight of total DNA nanocarriers)

**Table S4.** IC_50_ value of DOX in each group under different conditions.

|  | Normoxia (μg/mL) | Hypoxia (μg/mL) |
| --- | --- | --- |
| Free DOX | 2.22 | 9.71 |
| DA-DDF | 0.67 | 2.87 |
| DHA-DDF | 0.52 | 1.28 |
| DOX+US | 0.90 | 3.59 |
| DA-DDF+US | 0.39 | 0.98 |
| DHA-DDF+US | 0.33 | 0.54 |

**Table S5.** Primer sequences for real-time RT-PCR analyze.

| Gene | Forward Primer (5’→3’) | Reverse Primer (5’→3’) |
| --- | --- | --- |
| HIF-1α | ACCCATTCCTCATCCGTCAA | CTTCCGGCTCATAACCCATC |
| VEGF | GACCCTGGTGGACATCTTCCAGGA | GGTGAGAGGTCTAGTTCCCGA |
| 18s rRNA | CTTAGAGGGACAAGTGGCG | ACGCTGAGCCAGTCAGTGTA |


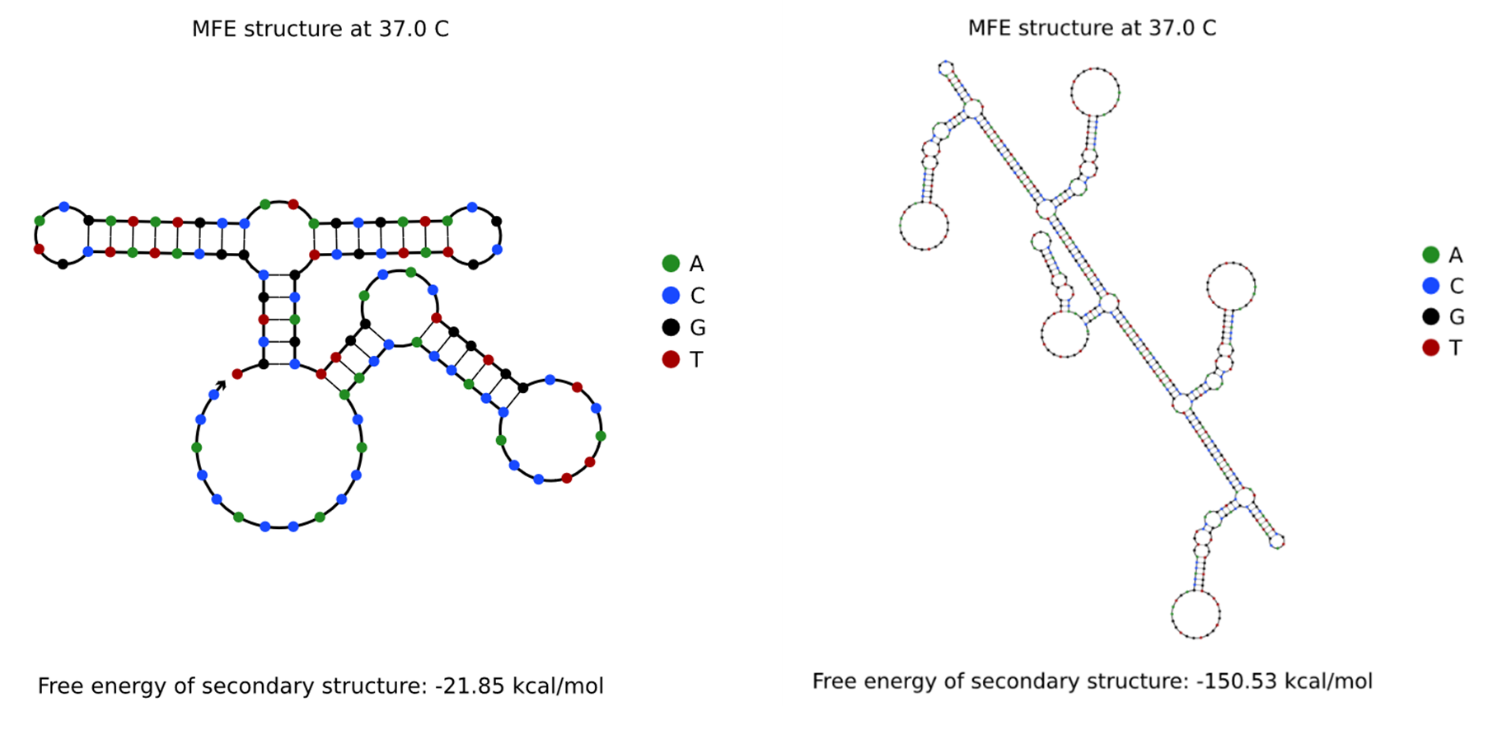


**Figure S1.** Predicted secondary structures of the linear DHA template (left) and pentamer RCA products (right) using the Nupack software with a parameter set at 37 °C.


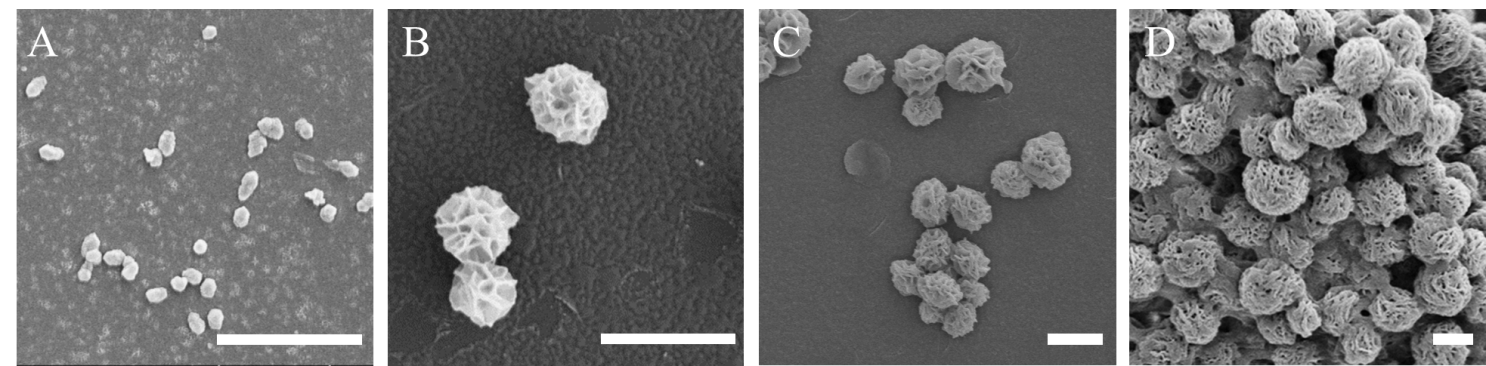


**Figure S2.** SEM images of DHA-DF obtained from RCA for 4 h (A), 8 h (B), 12 h (C), 16 h (D), scale bar: 1μm.


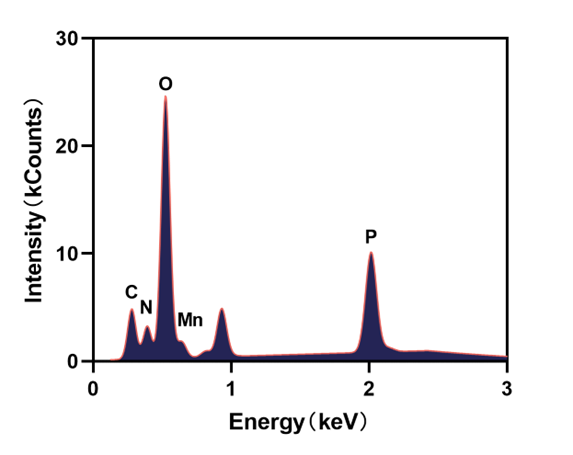


**Figure S3.** EDS analysis of DHA-DF.


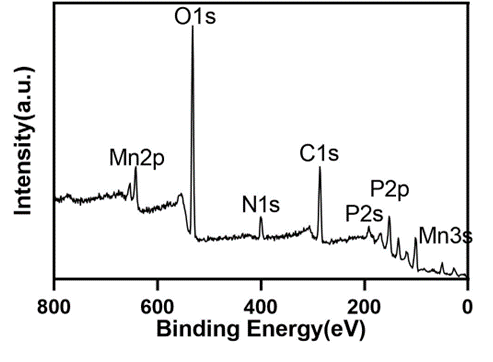


**Figure S4.** XPS spectra analysis of DHA-DF.


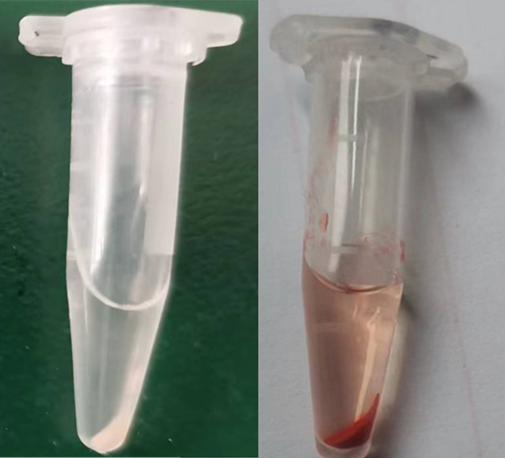


**Figure S5.** Images of DHA-DF (left) and DHA-DDF (right).


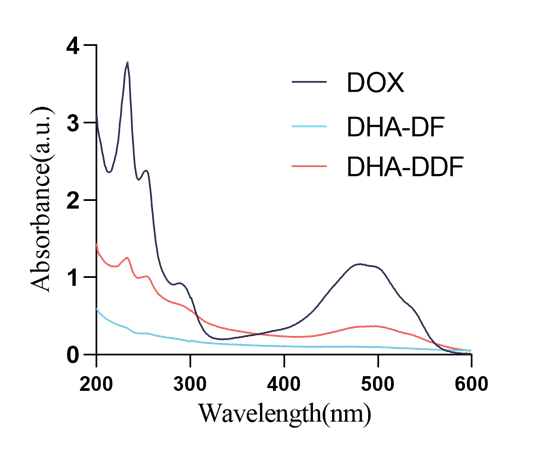


**Figure S6.** UV−vis absorption spectra of DOX, DHA-DF and DHA-DDF.


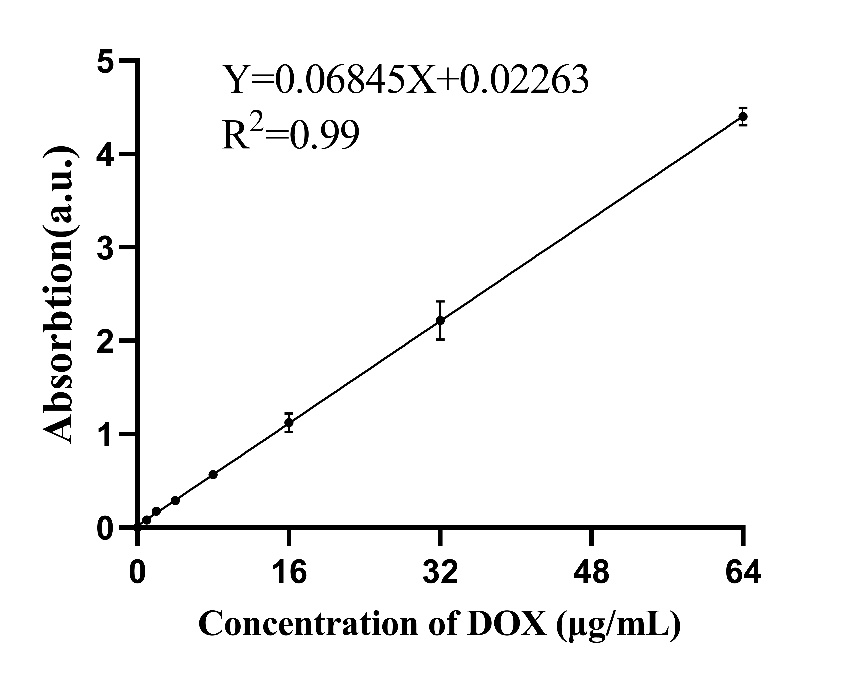


**Figure S7.** Standard curve of DOX. Error bars denote mean ± S.D. (n = 3)


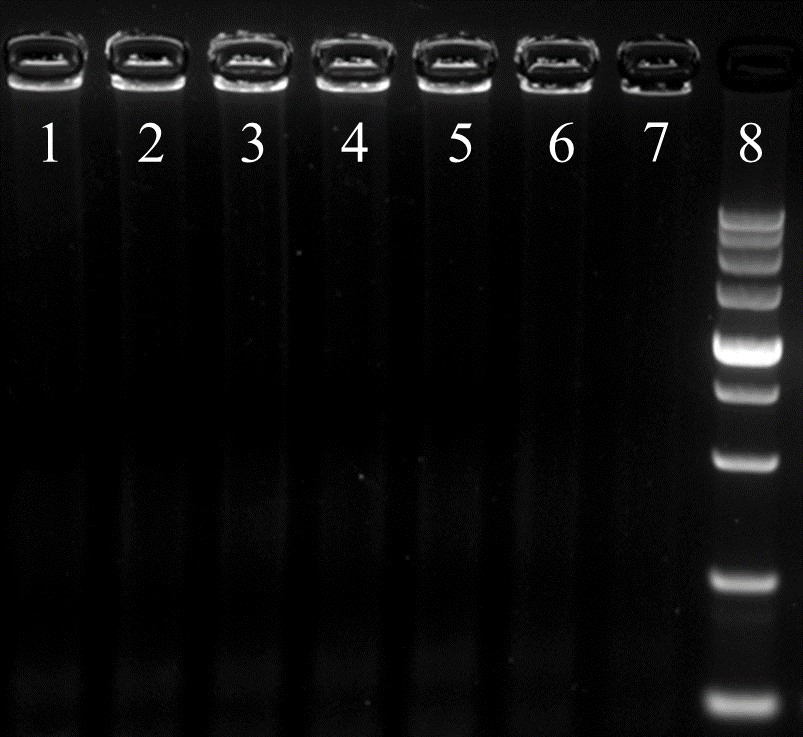


**Figure S8.** 2% agarose gel electrophoresis of DHA-DDF incubated with DMEM (10% serum plus 5 U/mL DNase I) for 0 h (Lane 1), 2 h (Lane 2), 4 h (Lane 3), 8 h (Lane 4), 1 d (Lane 5), 3d (Lane 6), 7 d (Lane 7).


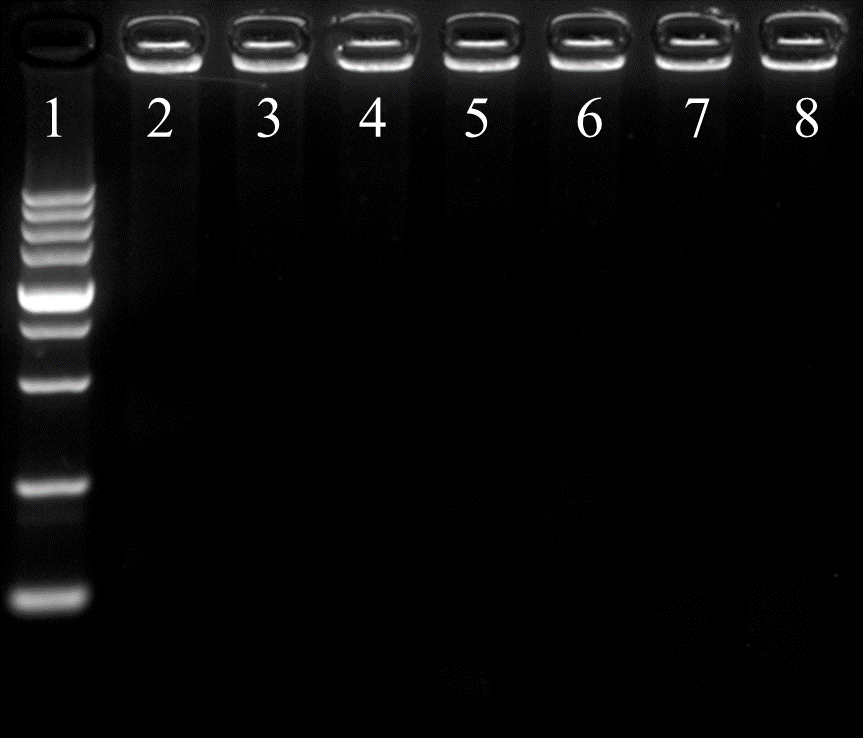


**Figure S9.** 2% agarose gel electrophoresis of DHA-DDF incubated with MES (pH = 5.5) for 0 h (Lane 2), 2 h (Lane 3), 4 h (Lane 4), 8 h (Lane 5), 1 d (Lane 6), 3d (Lane 7), 7 d (Lane 8).


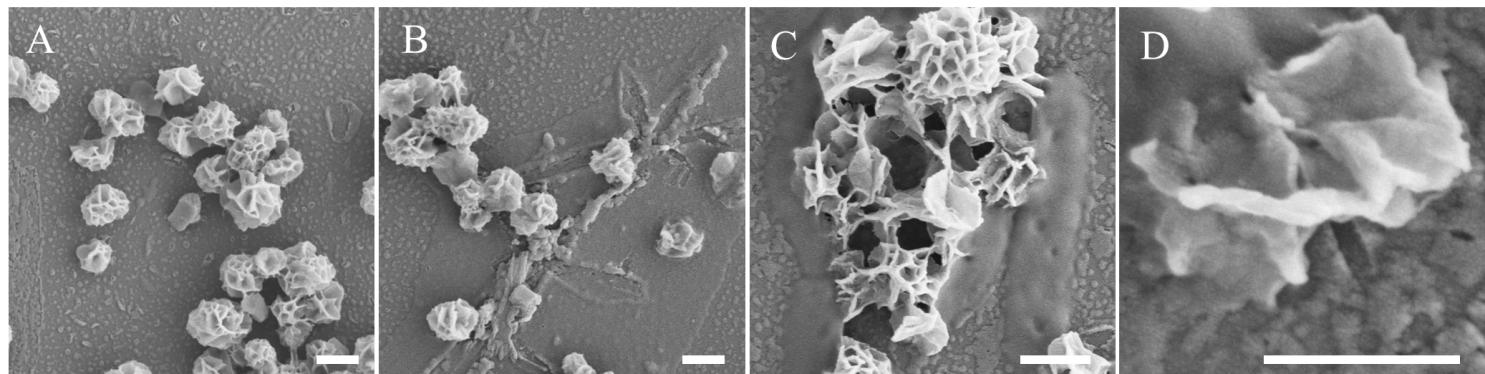
**Figure S10.** SEM images of DHA-DDF treated with FBS + DNase I for 8 h (A), 24 h (B), 3 d (C) and 7 d (D), scale bar: 500 nm.


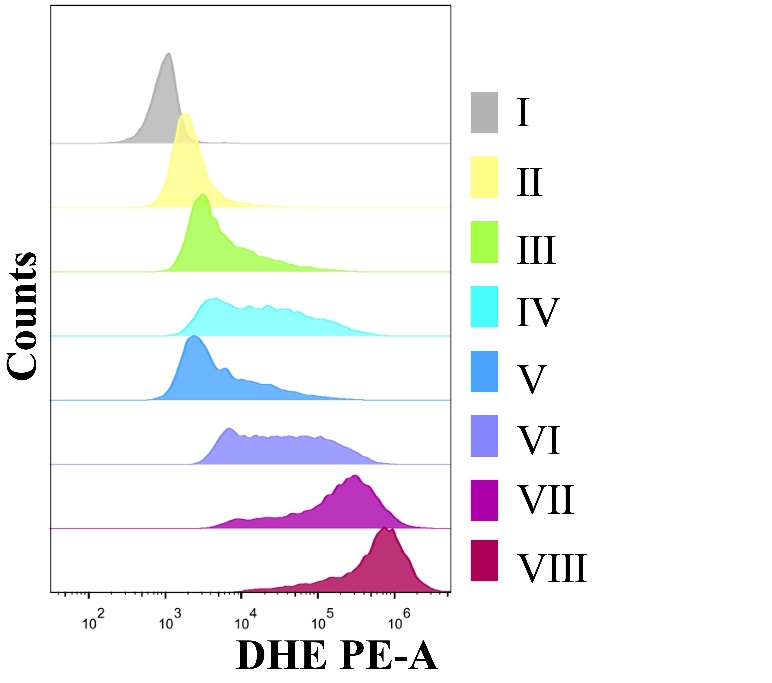


**Figure S11.** Flow cytometric analysis of ·O^2-^ generation after various treatment. I: PBS, II: DOX, III: DA-DDF, IV: DHA-DDF, V: PBS+US, VI: DOX+US, VII: DA-DDF+US, VIII: DHA-DDF+US.


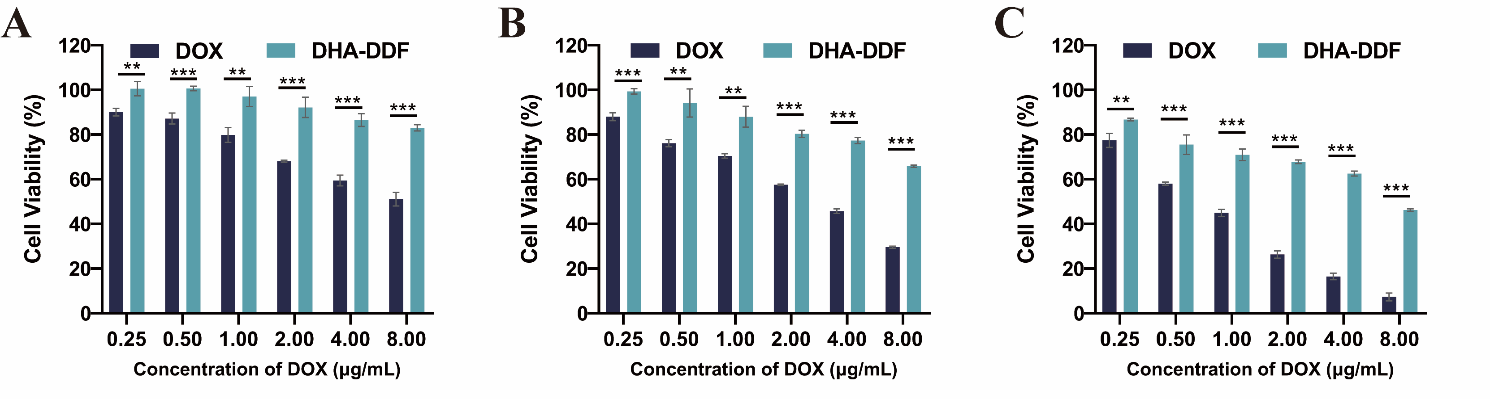


**Figure S12.** The activity of 16HBE cells treated with different concentrations of free DOX or DHA-DDF for 24 h (A), 48 h (B) and 72 h (C), respectively.


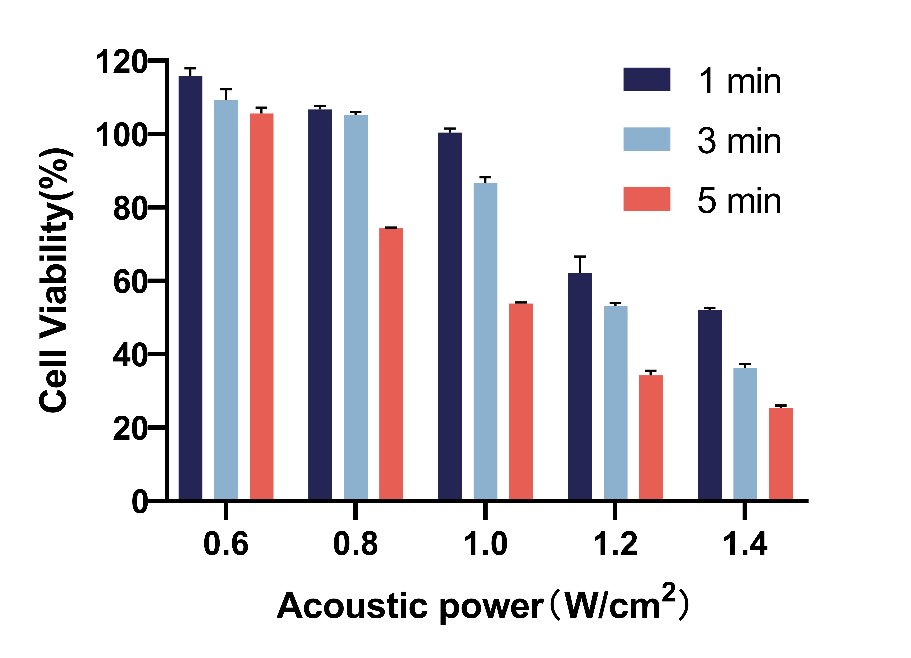


**Figure S13.** US dose screening *in vitro*.


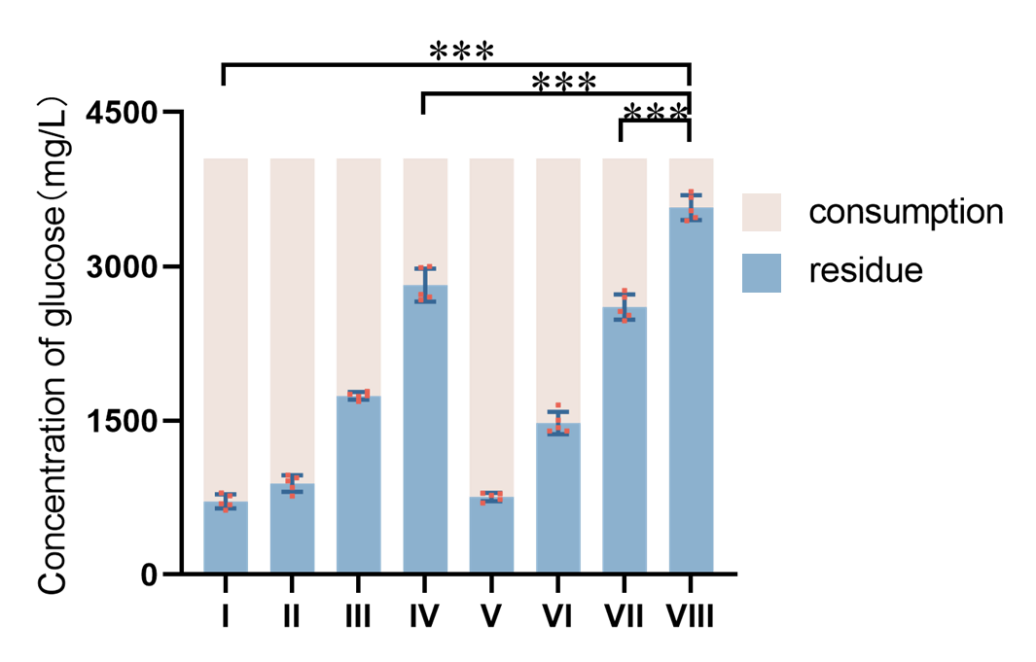


**Figure S14.** The concentration of glucose of DMEM culture medium after various treatment. I: PBS, II: DOX, III: DA-DDF, IV: DHA-DDF, V: PBS+US, VI: DOX+US, VII: DA-DDF+US, VIII: DHA-DDF+US.


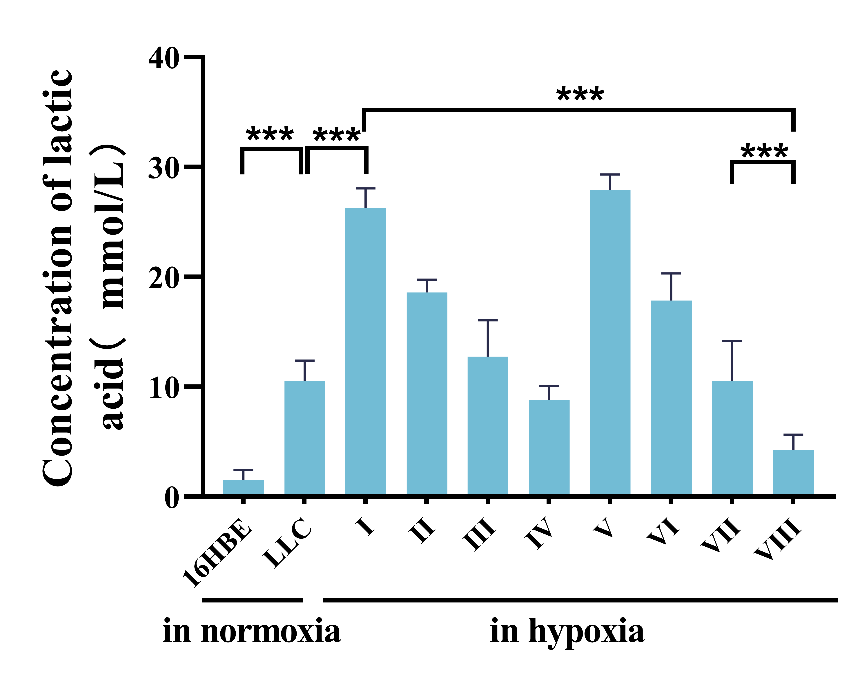


**Figure S15.** The concentration of lactic acid of DMEM culture medium in various conditions. I: PBS, II: DOX, III: DA-DDF, IV: DHA-DDF, V: PBS+US, VI: DOX+US, VII: DA-DDF+US, VIII: DHA-DDF+US.


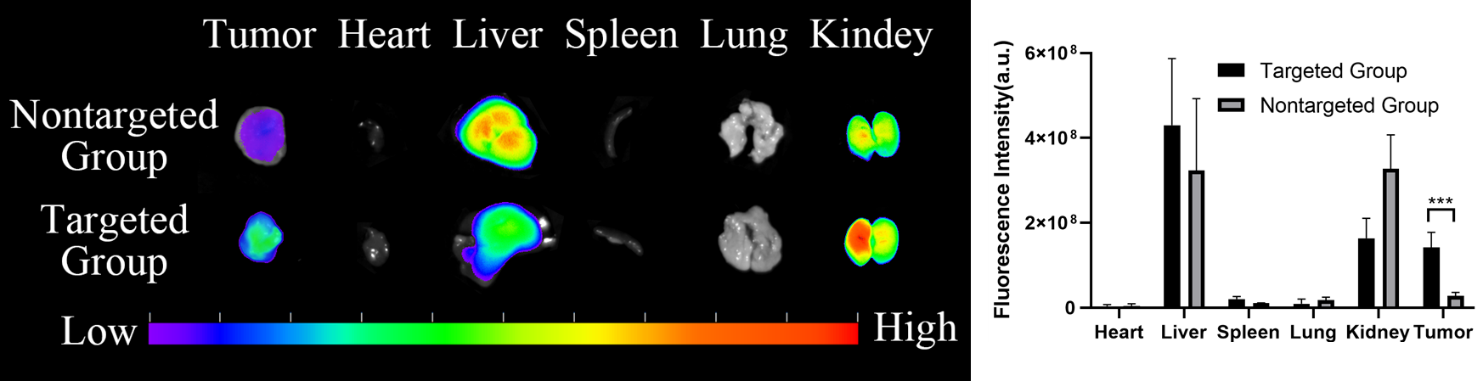


**Figure S16.** Fluorescence image and quantitative analysis of tumor tissues and major organs at 48 h after injecting Cy5-DH-DF (Nontargeted Group) and Cy5-DHA-DF (Targeted Group).


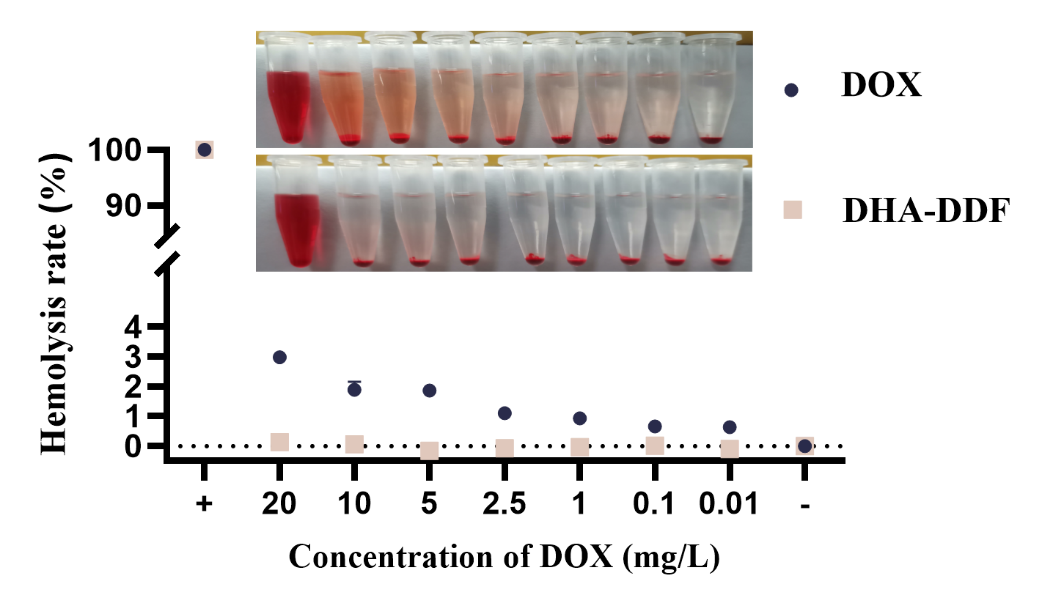


**Figure S17.** Hemolysis analysis of DOX and DHA-DDF at different concentration (0.01, 0.1, 1, 2.5, 5, 10 and 20 mg/L)


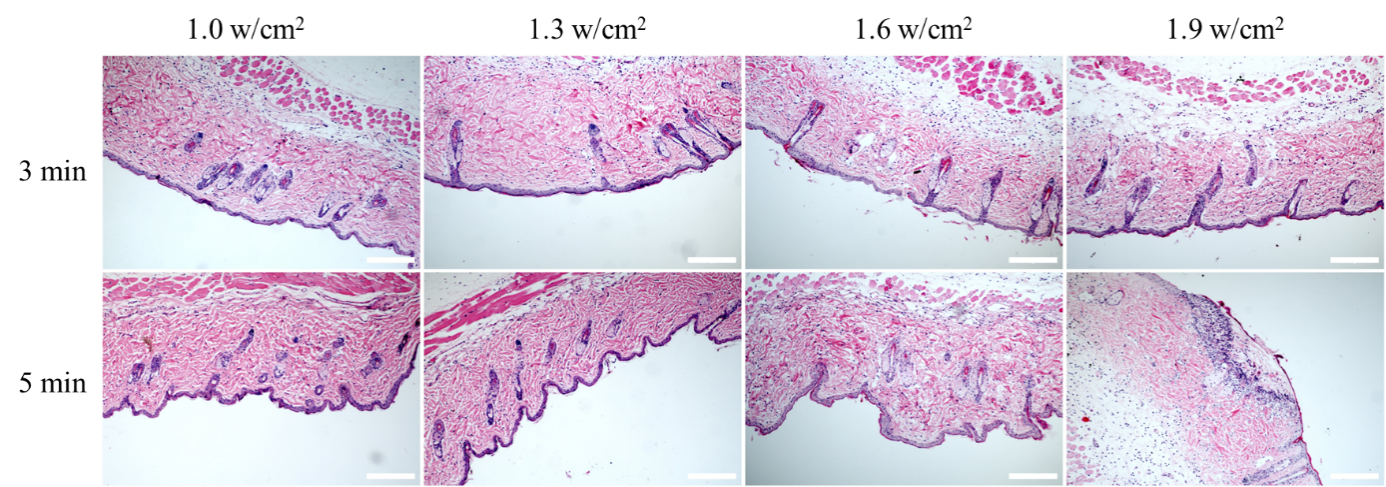


**Figure S18.** H&E pathological analysis of buttock skin tissues from C57BL/6 mice, scale bar: 200 μm.


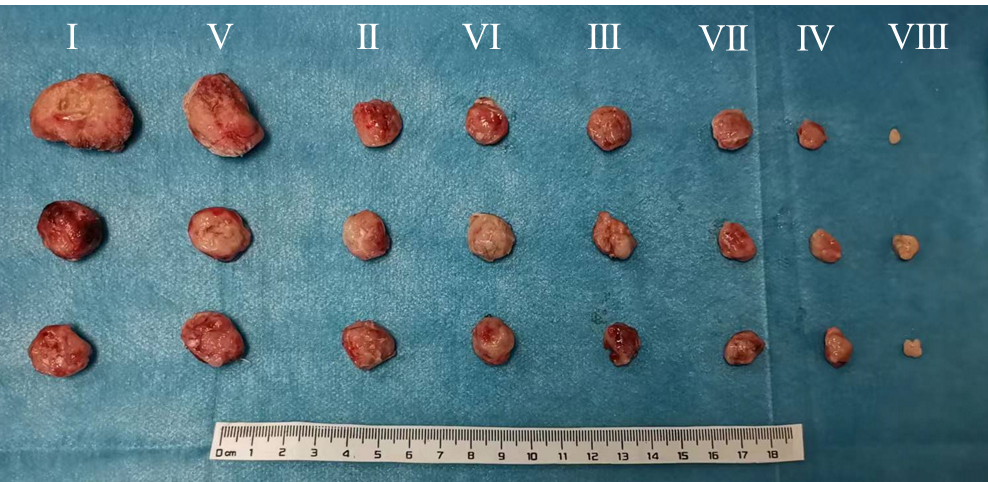


**Figure S19.** Photographs of *ex vivo* tumors after 14 days of different treatments. I: PBS, II: DOX, III: DA-DDF, IV: DHA-DDF, V: PBS+US, VI: DOX+US, VII: DA-DDF+US, VIII: DHA-DDF+US.


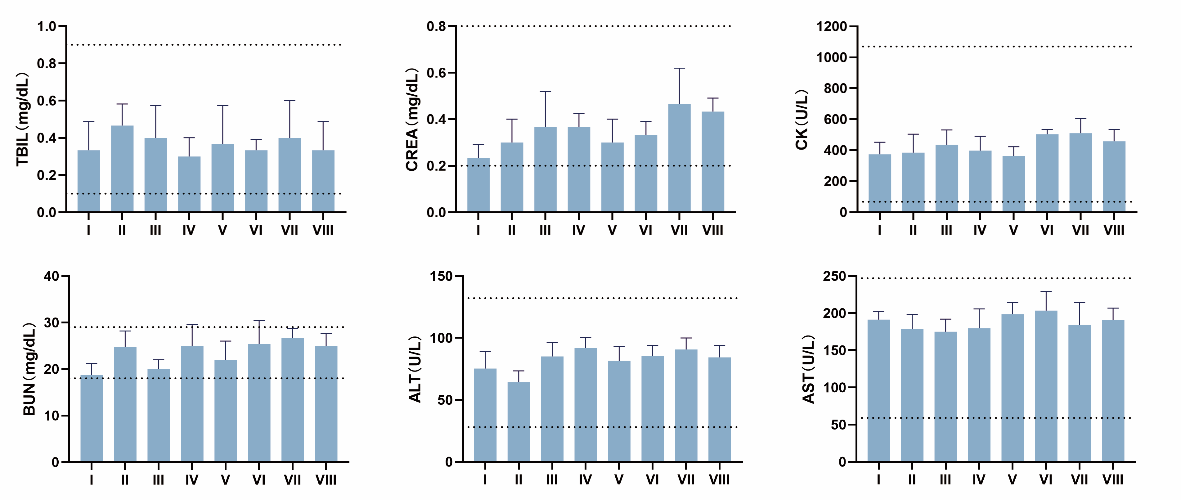


**Figure S20.** Blood biochemical analysis at 7 days post-treatment. (dotted lines represent the reference interval). I: PBS, II: DOX, III: DA-DDF, IV: DHA-DDF, V: PBS+US, VI: DOX+US, VII: DA-DDF+US, VIII: DHA-DDF+US.


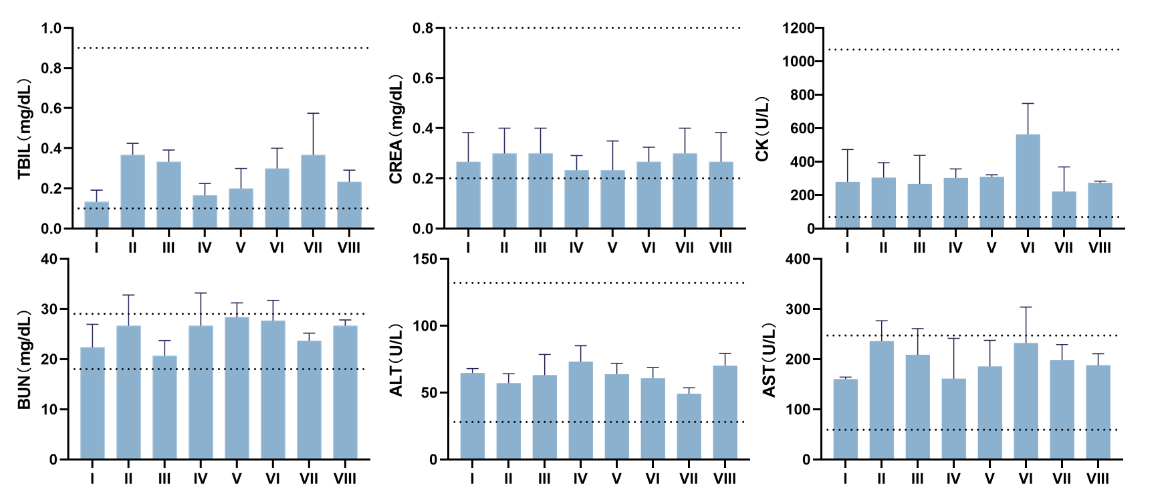


**Figure S21.** Blood biochemical analysis at 15 days post-treatment. (dotted lines represent the reference interval). I: PBS, II: DOX, III: DA-DDF, IV: DHA-DDF, V: PBS+US, VI: DOX+US, VII: DA-DDF+US, VIII: DHA-DDF+US.


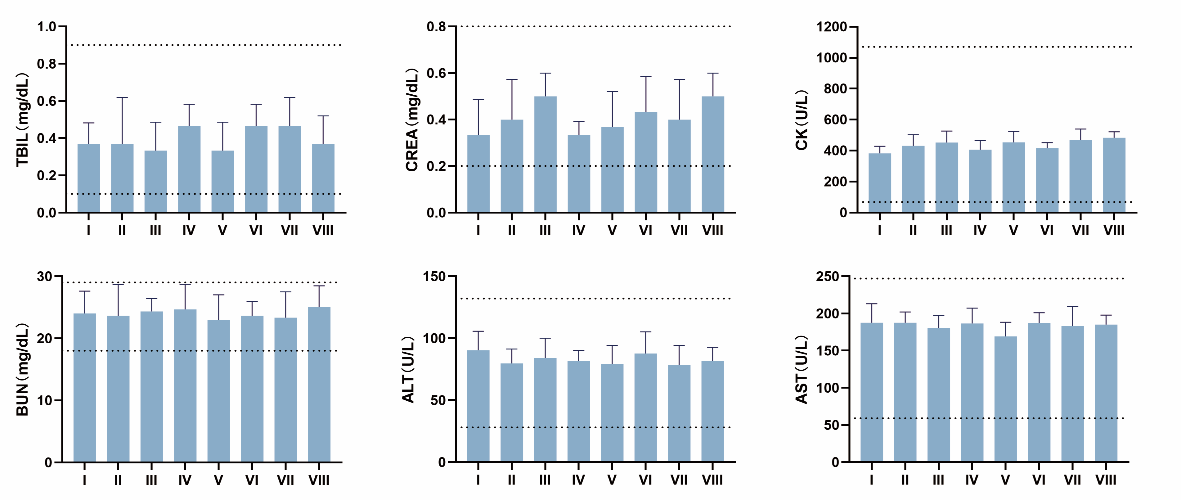


**Figure S22.** Blood biochemical analysis at 30 days post-treatment. (dotted lines represent the reference interval). I: PBS, II: DOX, III: DA-DDF, IV: DHA-DDF, V: PBS+US, VI: DOX+US, VII: DA-DDF+US, VIII: DHA-DDF+US.


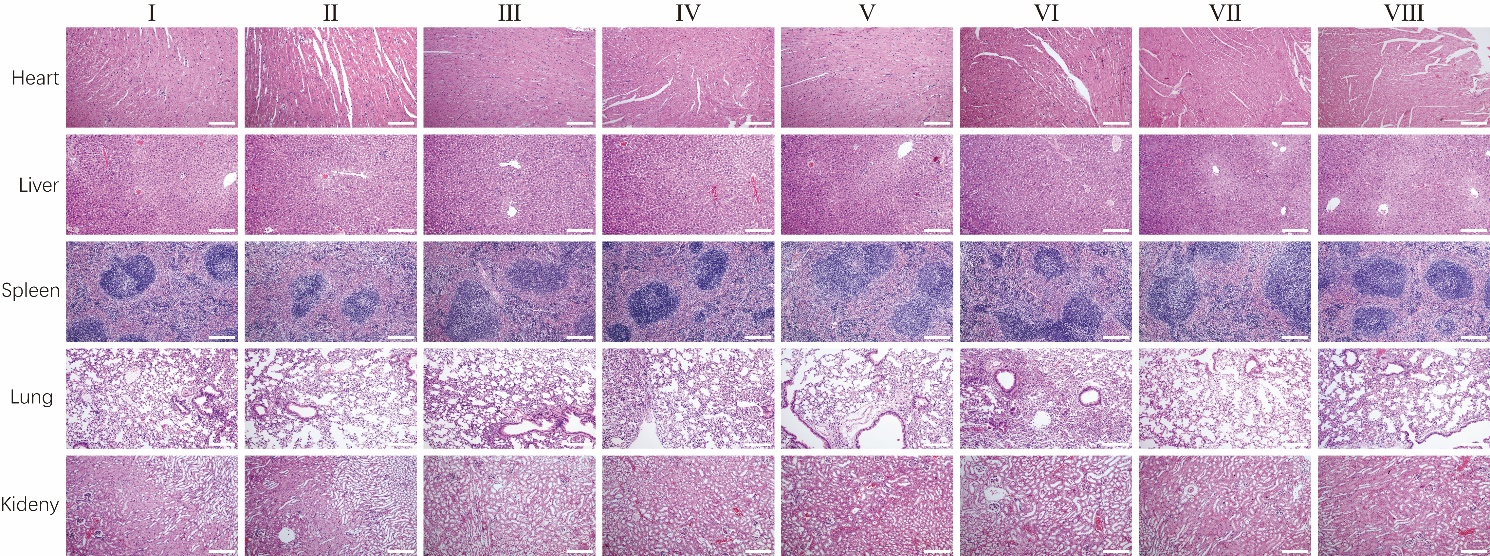


**Figure S23.** Histological analysis of major organs by H&E staining at 15 days post-treatment, scale bar: 100 μm. I: PBS, II: DOX, III: DA-DDF, IV: DHA-DDF, V: PBS+US, VI: DOX+US, VII: DA-DDF+US, VIII: DHA-DDF+US.


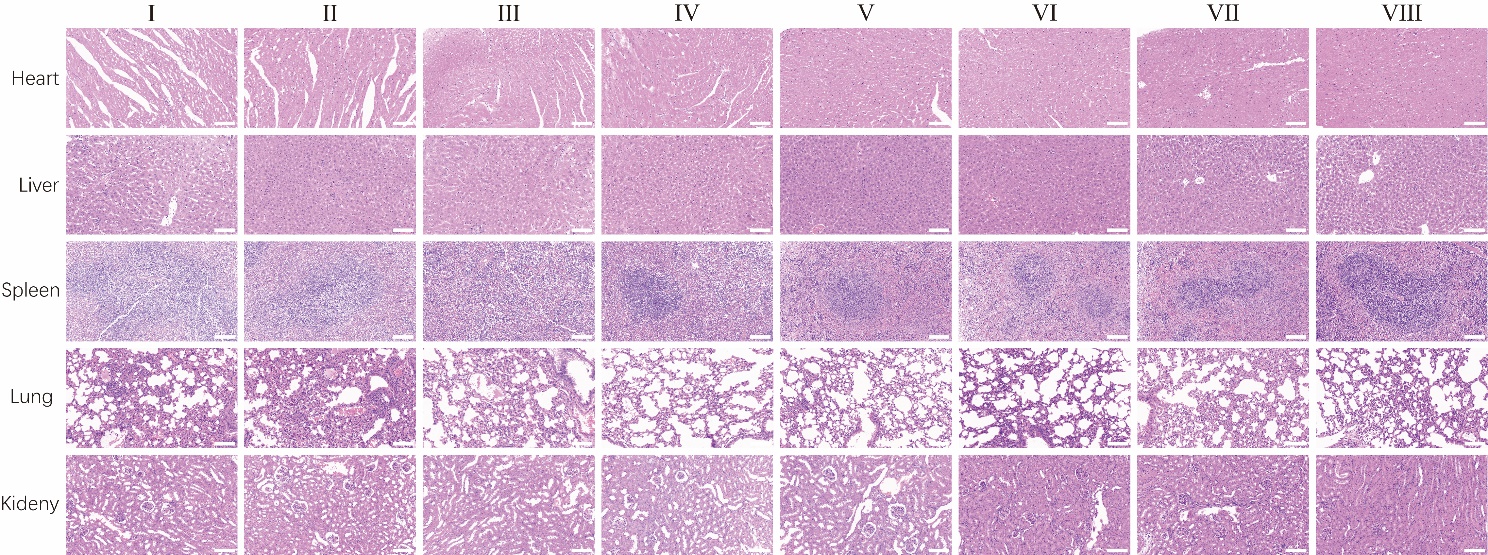


**Figure S24.** Histological analysis of major organs by H&E staining at 30 days post-treatment, scale bar: 100 μm. I: PBS, II: DOX, III: DA-DDF, IV: DHA-DDF, V: PBS+US, VI: DOX+US, VII: DA-DDF+US, VIII: DHA-DDF+US.


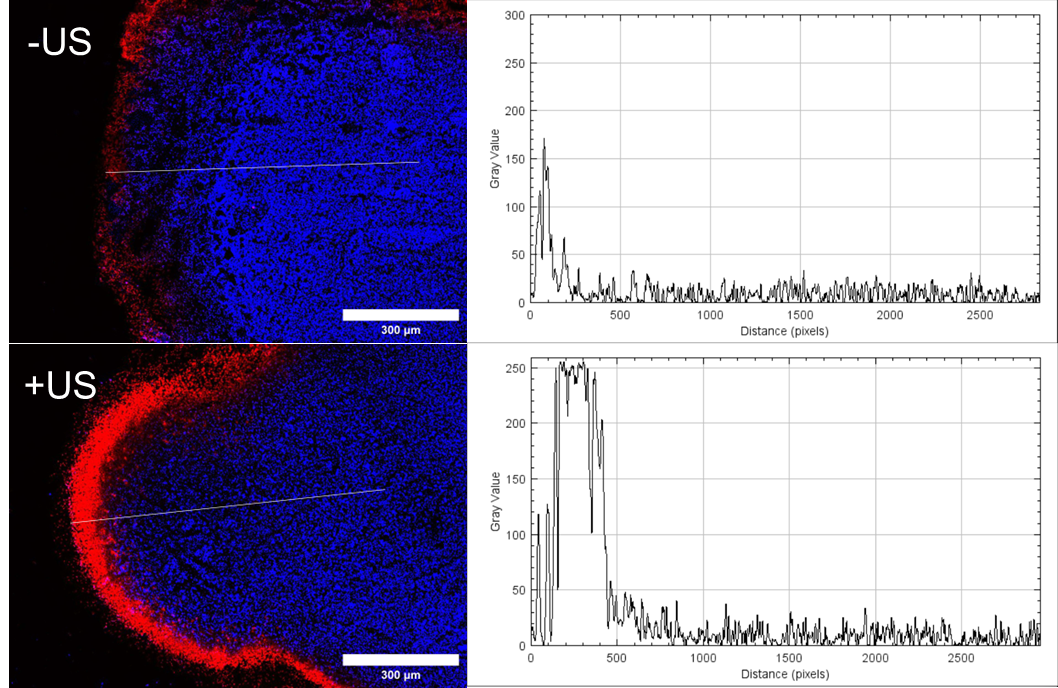


**Figure S25.** Analysis of ultrasound enhanced infiltration on *ex vivo* tumor.
